# Supplementary material for: ATRX Plays a Key Role in Maintaining Silencing at Interstitial Heterochromatic Loci and Imprinted Genes
Source: Cell Rep. 2015 Apr 9;11(3):405–18. doi: 10.1016/j.celrep.2015.03.036 (PMC4410944; doi:10.1016/j.celrep.2015.03.036)
Supplement: Document S1. Supplemental Experimental Procedures, Figures S1–S6, and Tables S1–S5 [file mmc1.pdf]

Cell Reports
Supplemental Information

ATRX Plays a Key Role in Maintaining Silencing at Interstitial Heterochromatic Loci and Imprinted Genes

Hsiao P.J. Voon, Jim R. Hughes, Christina Rode, Inti A. De La Rosa-Velázquez, Thomas Jenuwein, Robert Feil, Douglas R. Higgs, and Richard J. Gibbons

Figure S1

Related to Fig. 1

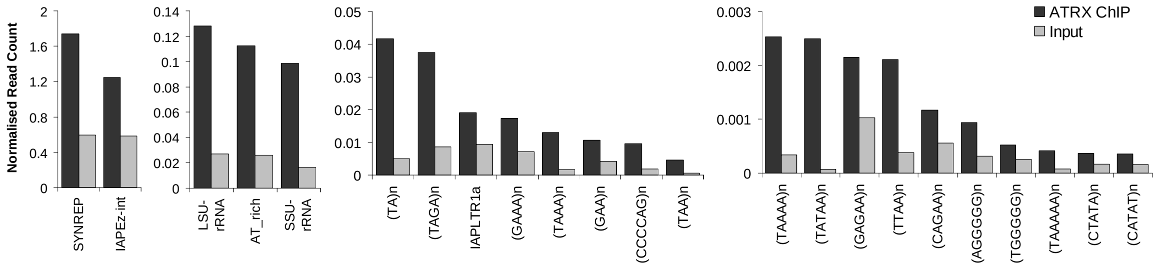

Figure S2
Related to Fig. 2

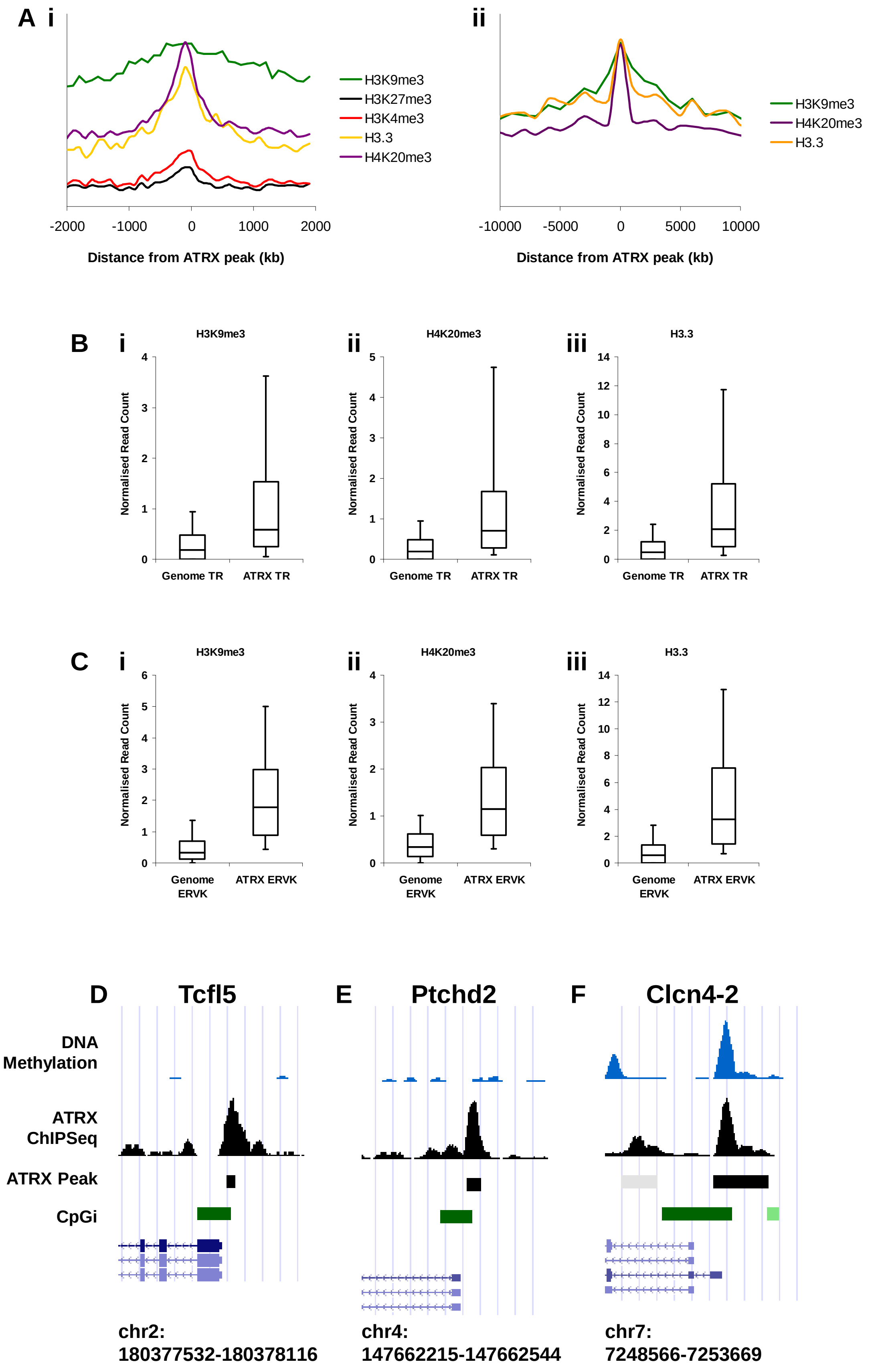

Figure S3

Related to Table 1

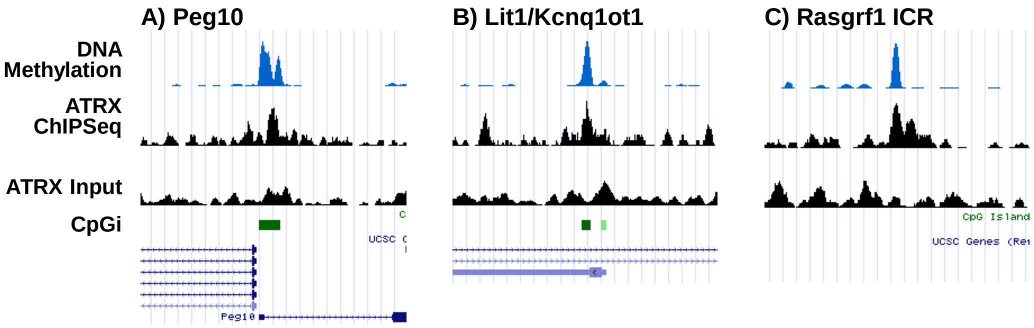

Figure S4

Related to Fig. 3

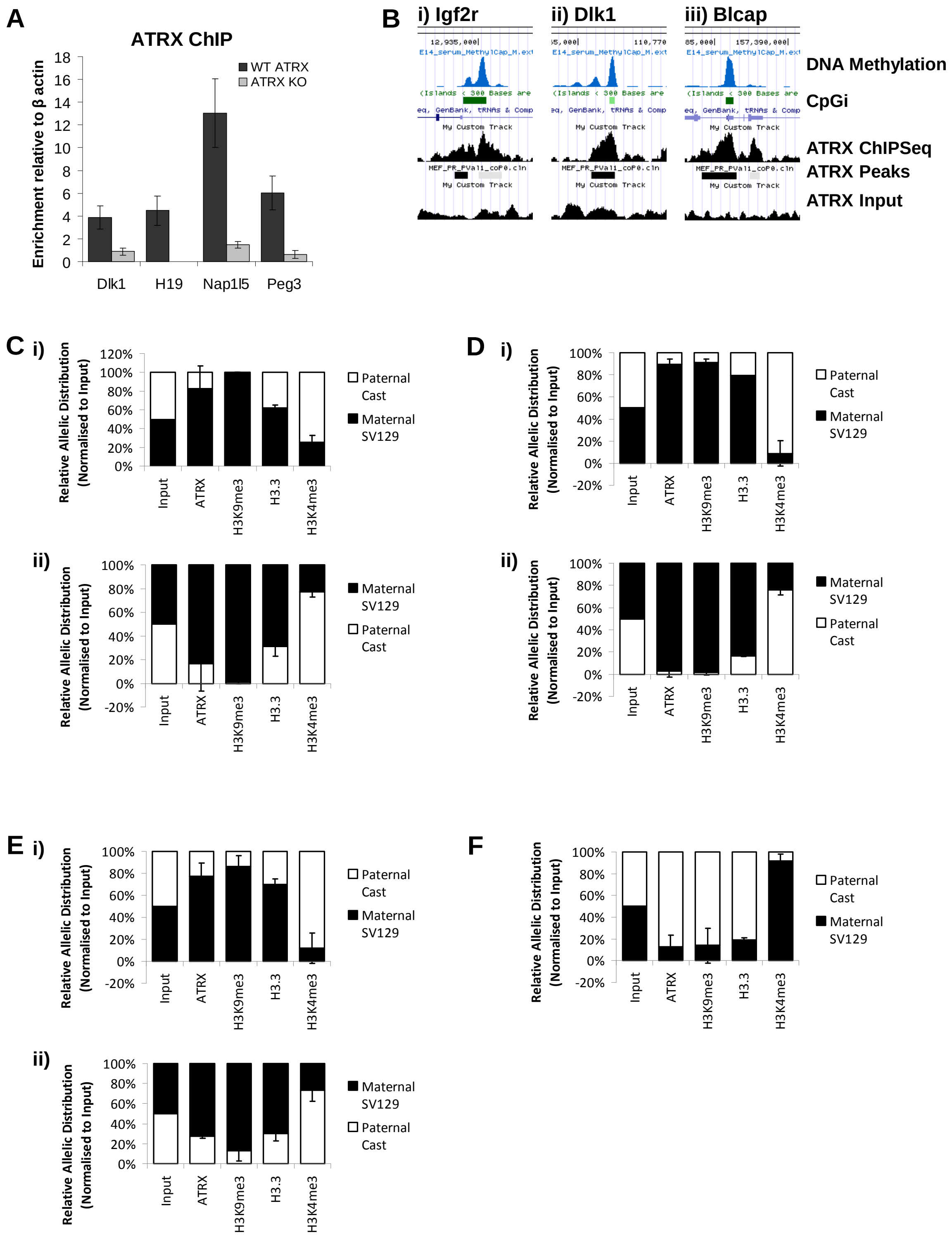

Figure S5

Related to Fig. 4

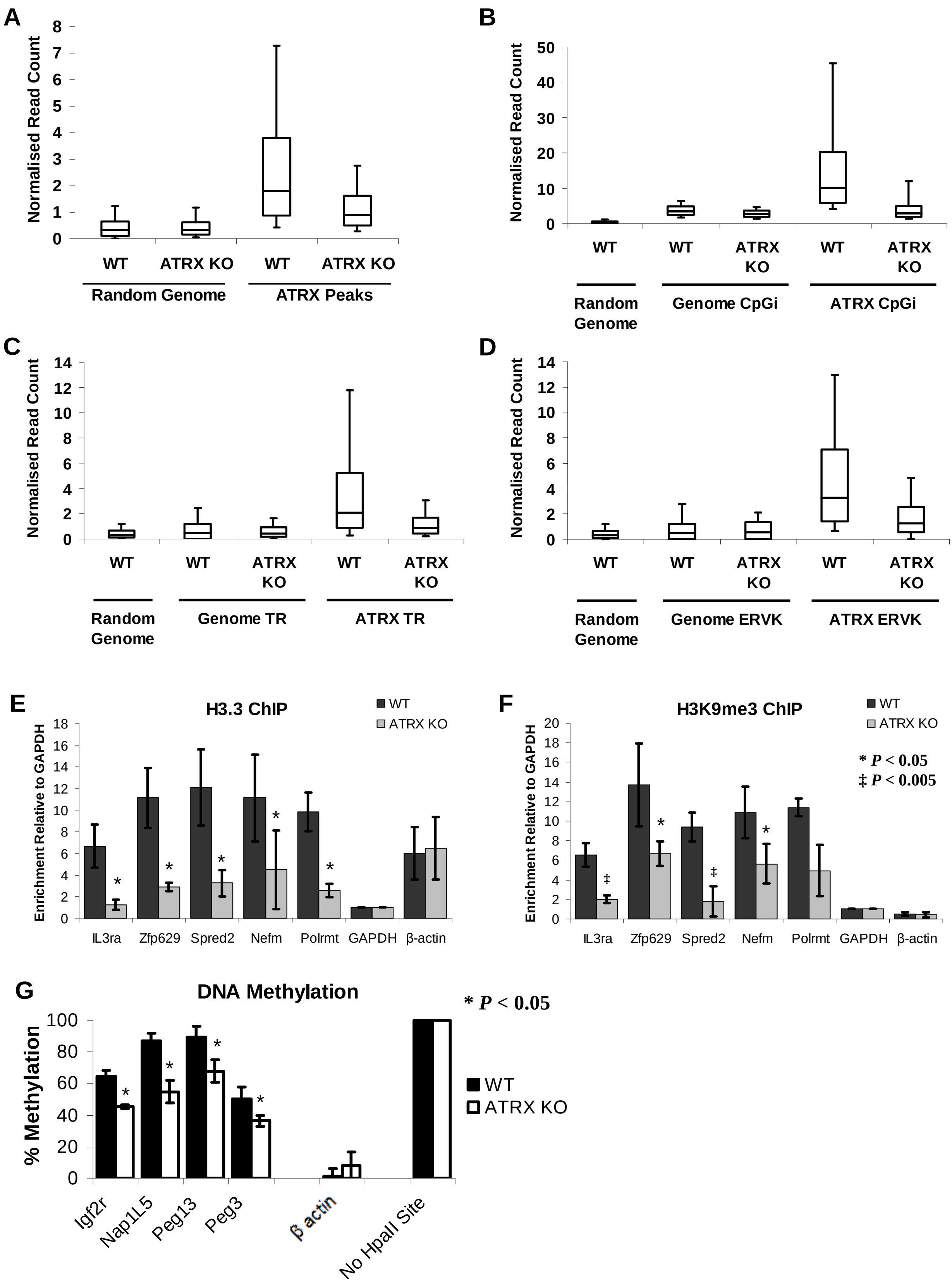

Figure S6

Related to Fig. 5

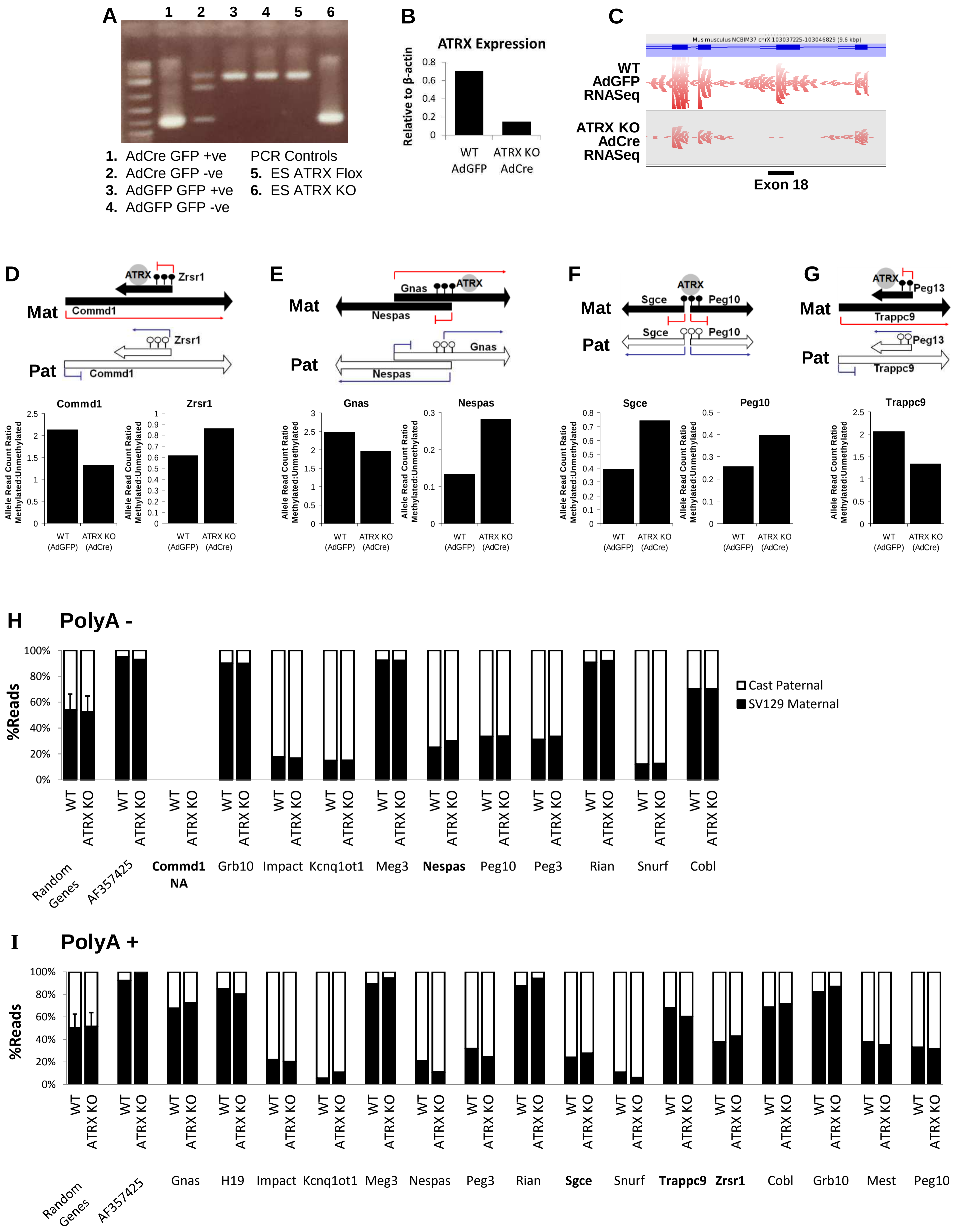

Figure S1, Related to Figure 1. ATRX is enriched at a number of repeats throughout the genome. ATRX ChIP-Seq reads were aligned against genomic repeats and compared against input enrichment after normalising for total read counts.

Figure S2, Related to Figure 2. All ATRX genomic targets are enriched for H3K9me3, H4K20me3 and H3.3. A) Pileup of various chromatin modifications in a **i)** 2 kb and **ii)** 10 kb window around ATRX peaks. ATRX bound regions are highly enriched for H3K9me3, H4K20me3 and H3.3. H3K9me3 enrichment is extended across a broader domain as seen in the 10 kb genomic window. **B)** Average normalised read counts of **i)** H3K9me3 **ii)** H4K20me3 and **iii)** H3.3 at ATRX bound tandem repeats (TR) compared to genomic TRs. **C)** Average normalised read counts of **i)** H3K9me3 **ii)** H4K20me3 and **iii)** H3.3 at ATRX bound ERVK repeats compared to genomic ERVK repeats. Boxes represent the 25th, median and 75th percentile. Whiskers represent the 90th and 10th percentile. ATRX bound TRs and ERVK repeats are enriched for H3K9me3, H4K20me3 and H3.3 compared to respective genomic controls. **D-F)** Examples of ATRX binding at promoter associated CGIs. ATRX binding profiles at **D)** *Tcfl5* **E)** *Ptchd2* and **F)** *Clcn4-2*. Regions where ATRX binding is annotated at promoter associated CGIs tend to show minimal overlap between ATRX peaks and CGIs.

Figure S3, Related to Table 1. Enrichment of ATRX at imprinted DMRs which are missed in peak calls. ATRX ChIP-seq profiles at the **A)** *Peg10* **B)** *Lit1/Kcnq1ot1* and **C)** *Rasgrf1* DMRs. ATRX input sequencing is shown for comparison. ChIP-seq and input tracks are shown on the same scale after normalising for total read counts.

Figure S4, Related to Figure 3. ATRX binds specifically to imprinted DMRs. A) ChIP-qPCR of ATRX at imprinted CpGi. ATRX ChIP was performed in ATRX KO cells as a negative control. Results represent the mean ± standard deviation of three independent experiments. **B)** Screenshots of ATRX ChIP-seq showing localisation specifically at methylated CGI in imprinted genes. **i)** Igf2r DMR **ii)** IG-DMR and **iii)** Blcap/Nnat DMR. **C-F)** Gel quantitation of allelic discrimination ChIP-PCR digests relative to input. Data represent the average of three independent experiments ± standard deviation. **C)** Peg13 digested with **i)** BtsCI **ii)** MscI. **D)** Peg3 digested with **i)** HaeIII **ii)** AvaII. **E)** Nap1L5 digested with **i)** HhaI **ii)** BstAPI and **F)** IG-DMR digested with BseRI.

Figure S5, Related to Figure 4. H3.3 enrichment is a general feature associated with ATRX binding and H3.3 enrichment is lost in ATRX KO cells. A) Average normalised read counts of H3.3 in WT vs ATRX KO cells at all ATRX binding sites across the genome compared to random genomic fragments. **B-D)** Average normalised read counts of H3.3 at major ATRX associated elements in the genome. The genomic average for **B)** CGIs, **C)** TRs and **D)** ERVKs were compared to respective ATRX bound elements in WT vs ATRX KO cells. All sites associated with ATRX enrichment show decreased H3.3 incorporation in ATRX KO cells compared WT cells. Boxes represent the 25th, median and 75th percentile. Whiskers represent the 90th and 10th percentile. ChIP qRT-PCR of **E)** H3.3 and **F)** H3K9me3 at ATRX bound intragenic CGI targets in WT and ATRX KO cells. Enrichment of both H3.3 and H3K9me3 at intragenic CGIs were lost in ATRX KO cells. Results represent the mean ± standard deviations of three independent experiments. **G)** DNA methylation at imprinted DMRs in WT and ATRX KO mouse ES cells. Results represent the mean ± standard deviations of three independent experiments (refer to supplemental methods).

Figure S6, Related to Figure 5. ATRX KO results in aberrant allelic expression of imprinted genes. A) Genotyping PCR of ATRX in GFP sorted cells transduced with AdGFP (WT) or AdCre (ATRX KO). **B)** qRT-PCR of ATRX expression in GFP sorted cells treated with AdGFP and AdCre. **C)** RNA-seq of WT AdGFP and ATRX KO AdCre cells. AdCre treated cells lack reads from exon 18. **D-G)** Ratio of methylated to unmethylated allelic reads from parental SNP informative reads at various imprinted loci. **D)** *Commd1/Zrsr1* **E)** *Gnas/Nespas* **F)** *Sgce/Peg10* and **G)** *Trappc9*. ATRX preferentially localises to the methylated maternal SV129 allele at all depicted loci. ATRX KO cells show disrupted allelic expression profiles compared to WT cells with SV129 maternal reads particularly affected. **H-I)** Differential allelic expression is reproducible in ATRX KO cells. An independent ATRX KO and RNA-seq experiment recapitulated aberrant allelic expression.

Table S1

Related to Fig. 2

Chr	Start	Stop	Promoter Status	Intragenic	Features
1	7386900	7388449	NonPromoter	Intragenic	
1	172993642	173014931	NonPromoter		
4	82158228	82159756	NonPromoter		
4	145424873	145426579	NonPromoter	Intragenic	
4	145723014	145724994	NonPromoter	Intragenic	
4	146559543	146561700	NonPromoter	Intragenic	
5	109977131	109988748	NonPromoter	Intragenic	
6	47693350	47694403	NonPromoter	Intragenic	
6	47727980	47729405	NonPromoter	Intragenic	
6	47971605	47978261	NonPromoter	Intragenic	
6	48543851	48548651	NonPromoter	Intragenic	
6	89784985	89785282	NonPromoter		
7	6081239	6086119	NonPromoter	Intragenic	
7	30577514	30579496	NonPromoter	Intragenic	
7	134750400	134759058	NonPromoter	Intragenic	
10	79195390	79204608	NonPromoter	Intragenic	
10	126949758	126950361	NonPromoter		
11	3023001	3024570	NonPromoter		
11	19917860	19924586	NonPromoter	Intragenic	
12	3234602	3241076	NonPromoter	Intragenic	
12	110764205	110768003	NonPromoter		Imprinted
13	47105262	47107083	NonPromoter	Intragenic	
13	120276362	120284312	NonPromoter	Intragenic	
14	15177661	15188428	NonPromoter	Intragenic	
14	52054268	52056141	NonPromoter	Intragenic	
19	5825902	5848706	NonPromoter		
19	61299571	61305080	NonPromoter	Intragenic	
X	166423462	166437580	NonPromoter	Intragenic	
2	157376955	157393095	Promoter	Intragenic	Imprinted
2	174120474	174124467	Promoter	Intragenic	Imprinted
6	30684845	30692843	Promoter		Imprinted
6	58854617	58859716	Promoter	Intragenic	Imprinted
7	6681284	6683762	Promoter	Intragenic	Imprinted
7	7230112	7232063	Promoter		Monoallelic
7	135828654	135835498	Promoter	Intragenic	Imprinted
10	12805933	12815343	Promoter	Intragenic	Imprinted
11	11918389	11930357	Promoter	Intragenic	Imprinted
11	22870617	22874230	Promoter	Intragenic	Imprinted
15	72639173	72641357	Promoter	Intragenic	Imprinted
17	12932143	12938087	Promoter	Intragenic	Imprinted
18	13130402	13133304	Promoter	Intragenic	Imprinted
17	39979942	39985774	rDNA		Monoallelic
1	90170106	90176974	Promoter		
2	180377532	180378116	Promoter		
3	21975167	21975472	Promoter		
4	120526901	120527621	Promoter		
4	147662215	147662544	Promoter		
5	124560770	124563038	Promoter		
7	7248566	7253669	Promoter		
8	4677024	4680280	Promoter		
8	19782933	19787537	Promoter		
8	19891139	19893565	Promoter		
10	80644767	80645797	Promoter		
12	18139974	18142499	Promoter		
14	8623565	8624031	Promoter		
15	88906698	88906897	Promoter		

Table S2

Related to Fig. 3

		Size	Enzyme	SV129	Cast
Nap1L5_allCh_fwd	TCACCTGTCAGCTTGCTGTGCT	190 bp	HhaI	87, 55, 48 bp	142, 48 bp
Nap1L5_allCh_rev	GCCCCTGCTCGCCAAGATCC		BstAPI	190 bp	131, 60 bp
Peg13_allCh_fwd	GCGGCAGTGTCGCAGGTCTT	187 bp	BtsCI	126, 61 bp	187 bp
Peg13_allCh_rev	CCGAAGAGGACCCGGTGGCT		MscI	187 bp	111, 76 bp
Peg3_allch_fwd	GCCGCTCCGTGGTGATTCCC	154 bp	AvaII	154 bp	90, 64 bp
Peg3_allch_rev2	GCATGCACCCTCTTAGATACCGTCT		HaeIII	90, 64 bp	154 bp
Dlk1_allch_fwd2	GCCGTTCGCTATGAACTACCGCT	174 bp	BseRI	130, 44 bp	174 bp
Dlk1_allch_rev2	GTTCGCGGCACGCGTACACA				

Table S3

Related to Fig. 4

	Fwd	Rev
Dlk1	TGTGCCAGAGCAGTGCTACACG	GCCATGGCACAACTACACAGGGT
H19	CGACCACTGAGGCATAGCGGC	TGTCCAAGGGCCAAAGTTCGGG
Nap15L	CATGGCCGACCCCGAGAAGC	CGGCATCTTCGCCACCCTGG
Peg3	CACCGCCACTGCGGCAAAAC	GGCTGGCAGGGTCTTCGCAA
Plagl1	CCAGCGAGGGCTGAGTTGCC	GGCCTGGAAGGAAGCGTGCA
IL3ra	TCCCACCCTCGCGATGTGACT	GCACAAACCCTCATGGGGTGGC
Zfp629	TTGGCTCCTGGTGGCGGAGT	GCCCGGAGAGCTCAGGGTGA
Spred2	TGAAGACTACCGGCACGCGC	CCCTTGGCGAAGCGCACGTA
Nefm	TGGGGGCCTCGACTTTGGTCT	CAGGAAGCATCACCGGGCCT
Polrmt	CGGCCGCGGAAGTCCATGTT	CGCAGCGAGGCCCTGTATCG
β-actin	ACACCCGCCACCAGGTAAGCA	CCTGCAGTGAGGTACTAGCCACGA
GAPDH	TTGCTGTTGAAGTCGCAGGAGAC	GGCATCTTGGGCTACACTGAGGAC

	Fwd	Rev
Igf2r_Me	CCAAAGGTTCGGAGGGTTTT	ACTTGGCATAACCAGAATCACAG
Nap1L5_Me	GCAGGGGCTTGTAGATATCGTT	AGAGCTTGCCCAATTCCGTG
Peg13_Me	ATCGTCTACATAGCACCAGCG	TCTGTGACCACGAACCGAAG
Peg3_Me	TAGTGCACCCACACTGAACC	GACGAGCATCGGAGGAGAAG
β-actin	ACACCCGCCACCAGGTAAGCA	CCTGCAGTGAGGTACTAGCCACGA
Nap1L5 NoHpaII	CATGGCCGACCCCGAGAAGC	CGGCATCTTCGCCACCCTGG

Table S4

Related to Fig. 5

A

			Mapped Reads	Total mapped reads
PolyA +	AdGFP	Fwd reads	15042599	31835329
		Rev reads	16792730	
	AdCre	Fwd reads	12668012	25864638
		Rev reads	13196626	
PolyA -	AdGFP	Fwd reads	9242629	19867417
		Rev reads	10624788	
	AdCre	Fwd reads	9299015	19892878
		Rev reads	10593863	

B

1500 random genes		No. of Informative Genes	Total Informative Reads	Reads Mapped to SV129 Maternal Genome	Reads Mapped to Castaneus Paternal Genome
PolyA+	WT (AdGFP)	452	111323	56855	54468
	ATRX KO (AdCre)	438	109409	56352	53057
PolyA-	WT (AdGFP)	261	40432	22223	18209
	ATRX KO (AdCre)	253	38138	20695	17443

C

	Genes with >50 informative SNP reads	Genes with >66% expected parental skewing	Genes with moderate skewing towards expected allele (>60% and <66%)	Genes skewed against expected parental allele	Biallelic Expression
PolyA +	33	12	4	3	14
PolyA -	26	11	1	4	10

Table S5

Related to Fig. 2-4

	Series	Sample ID	Contact name	Cell type	Antibody
**ATRX**	GSE22162	GSM551138	Richard J Gibbons	Mouse ES	ATRX
**H3K9me3**	GSE57092	GSM1375155	Sarah Diehl	Mouse ES	H3K9me3
**H4K20me3**	GSE26680	GSM656527	Richard A Young	Mouse ES	H4K20me3
**DNA methylation**	GSE28254	GSM821397, GSM821398	Arjen Brinkman, Stunnenberg HG	Mouse ES	Medium and high MethylCap
**H3K4me3**	GSE12241	GSM307618	Tarjei S Mikkelsen	Mouse ES	H3K4me3
**ESHyb H3K4me3**	GSE12241	GSM307605	Tarjei S Mikkelsen	Mouse ESHyb	H3K4me3
**ESHyb H3.3**	GSE16893	GSM487542	Aaron D Goldberg	Mouse ESHyb	HA (H3.3)

Table S1, Related to Figure 2. Features associated with all ATRX bound CGIs in mouse ES cells. ATRX CGIs located within 1 kb of the TSS of a known gene were annotated as promoters. ATRX CGIs which overlapped a known gene but located greater than 1 kb away from a TSS were annotated as intragenic. ATRX CGIs associated with imprinted genes or known monoallelic genes are also shown.

Table S2, Related to Figure 3. List of allelic ChIP primers used in this study.

Table S3, Related to Figure 4. List of qRT-PCR ChIP and DNA methylation primers used in this study.

Table S4, Related to Figure 5. Data related to allelic RNA-seq. A) Total mapped reads in PolyA+ and PolyA- RNA-seq. **B)** Informative SNP reads mapped to SV129 and Castaneus genomes across 1500 random genes. **C)** Allelic expression skewing in imprinted genes with greater than 50 informative SNP reads.

Table S5. List of GEO datasets used throughout this study.

Supplemental Experimental Procedures

ChIP-seq Analysis

Random genomic fragments were generated using Random Genome Fragments on RSA-tools (Thomas-Chollier et al., 2011) using ATRX peaks as a template. Histone modifications were quantitated under random genomic fragments in Seqmonk after normalising for read count and fragment length. Regions were considered enriched for a particular modification if ChIP reads exceeded read counts of the 75th percentile of random fragments. Genome annotations (CGI, TR, ERV) were downloaded from the UCSC Table Browser (mm9 build) (http://genome.ucsc.edu/) (Karolchik et al., 2004) and overlaps were performed in Seqmonk. Where peaks overlapped with multiple features, regions were preferentially annotated in descending order as CGIs, TRs and ERVKs.

DNA Methylation Assay

Genomic DNA was extracted from WT and ATRX KO mouse ES cells and digested with either Msp I or Hpa II, a methylation sensitive isoschizomer. DNA digested with Hpa II were subjected to qRT-PCR with primers which flank an Msp I/Hpa II recognition site at imprinted DMRs. Undigested genomic DNA was combined with Msp I digested DNA in known ratios to obtain a standard curve. Hpa II digested products were plotted against respective curves and normalised to an amplicon which lacked an Msp I/Hpa II recognition site to obtain a methylation ratio. A list of primers used for this study can be found in Table S3.
