# Supplementary material for: ATRX Plays a Key Role in Maintaining Silencing at Interstitial Heterochromatic Loci and Imprinted Genes
Source: Cell Rep. 2015 Apr 9;11(3):405–18. doi: 10.1016/j.celrep.2015.03.036 (PMC4410944; doi:10.1016/j.celrep.2015.03.036)
Supplement: Document S2. Article plus Supplemental Information [file mmc2.pdf]

Cell Reports

Article

ATRX Plays a Key Role in Maintaining Silencing at Interstitial Heterochromatic Loci and Imprinted Genes

Graphical Abstract

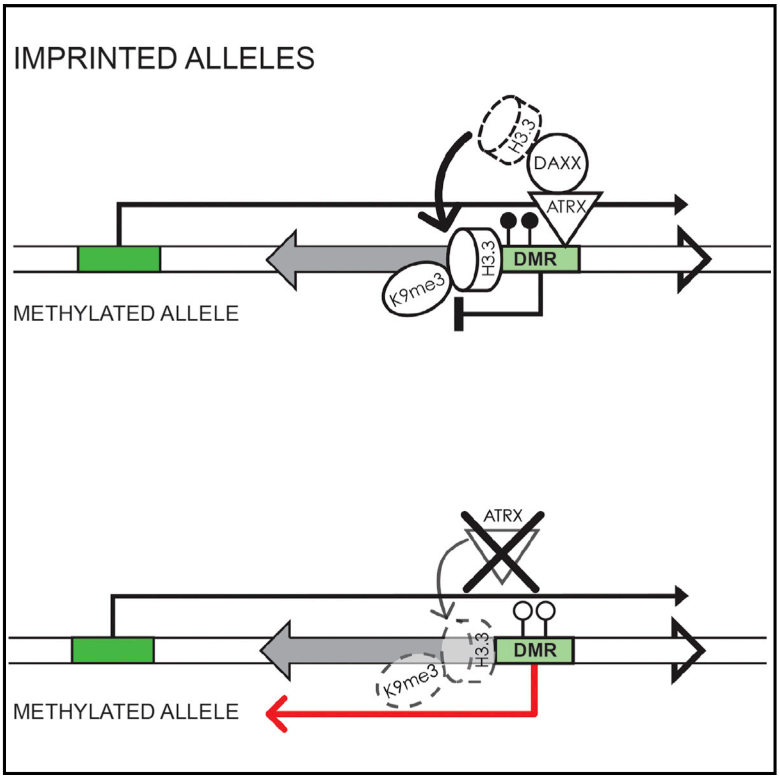

Authors

Hsiao P.J. Voon, Jim R. Hughes, ..., Douglas R. Higgs, Richard J. Gibbons

Correspondence

richard.gibbons@ndcls.ox.ac.uk

In Brief

Voon et al. demonstrate that ATRX deposits the histone variant H3.3 at the methylated allele of imprinted differentially methylated regions. ATRX knockout leads to the loss of H3.3 and the H3K9me3 heterochromatin modification at these regions, suggesting a unique role for H3.3 in maintaining heterochromatin silencing.

Highlights

- ATRX deposits H3.3 at heterochromatin throughout the genome
- ATRX and H3.3 preferentially bind the methylated allele of imprinted DMRs
- H3.3 deposition at imprinted DMRs is dependent on ATRX
- Loss of ATRX/H3.3 leads to loss of H3K9me3 modification at imprinted DMRs

Accession Numbers

GSE59271

Voon et al., 2015, Cell Reports *11*, 405–418
April 21, 2015 ©2015 The Authors
http://dx.doi.org/10.1016/j.celrep.2015.03.036

Cell Reports
Article

OPEN ACCESS
CellPress

ATRX Plays a Key Role in Maintaining Silencing at Interstitial Heterochromatic Loci and Imprinted Genes

Hsiao P.J. Voon,[1] Jim R. Hughes,[1] Christina Rode,[1] Inti A. De La Rosa-Velázquez,[2] Thomas Jenuwein,[2] Robert Feil,[3] Douglas R. Higgs,[1] and Richard J. Gibbons[1,*]
[1]MRC Molecular Haematology Unit, Weatherall Institute of Molecular Medicine, University of Oxford, Oxford OX3 9DS, UK
[2]Department of Epigenetics, Max Planck Institute of Immunobiology and Epigenetics, Freiburg 79108, Germany
[3]Institute of Molecular Genetics of Montpellier (IGMM), Centre National de la Recherche Scientifique (CNRS), 34293 Montpellier, France
*Correspondence: richard.gibbons@ndcls.ox.ac.uk
http://dx.doi.org/10.1016/j.celrep.2015.03.036
This is an open access article under the CC BY license (http://creativecommons.org/licenses/by/4.0/).

SUMMARY

Histone H3.3 is a replication-independent histone variant, which replaces histones that are turned over throughout the entire cell cycle. H3.3 deposition at euchromatin is dependent on HIRA, whereas ATRX/Daxx deposits H3.3 at pericentric heterochromatin and telomeres. The role of H3.3 at heterochromatic regions is unknown, but mutations in the ATRX/Daxx/H3.3 pathway are linked to aberrant telomere lengthening in certain cancers. In this study, we show that ATRX-dependent deposition of H3.3 is not limited to pericentric heterochromatin and telomeres but also occurs at heterochromatic sites throughout the genome. Notably, ATRX/H3.3 specifically localizes to silenced imprinted alleles in mouse ESCs. ATRX KO cells failed to deposit H3.3 at these sites, leading to loss of the H3K9me3 heterochromatin modification, loss of repression, and aberrant allelic expression. We propose a model whereby ATRX-dependent deposition of H3.3 into heterochromatin is normally required to maintain the memory of silencing at imprinted loci.

INTRODUCTION

In mammalian cells, DNA is packaged into chromatin, which can be differentially modified in association with different transcriptional states. Modifications associated with transcribed euchromatin include histone acetylation, H3K4me3 at promoters, and H3K36me3 within the gene body. The reversible gene silencing at facultative heterochromatin is mediated by H3K27me3 and the Polycomb group proteins, while permanent silencing of repetitive constitutive heterochromatin is linked to DNA methylation, H3K9me3, and H4K20me3. These epigenetic modifications are thought to contribute to gene regulation and to provide a template for cellular identity, heritable through cell divisions.

As modified histones are displaced from DNA during replication, the maintenance of chromatin states is dependent on the rapid reassembly of chromatin onto newly replicated DNA (Jasencakova et al., 2010). The reassembled chromatin is composed of a mixture of recycled, modified histones and naive histones (Groth et al., 2007; Jackson, 1988) newly synthesized during the S phase. The preservation of chromatin modifications in daughter cells theoretically could be accomplished by chromatin modifiers recognizing specific marks on recycled histones and imparting these same marks on adjacent newly synthesized histones (Abmayr and Workman, 2012; Groth et al., 2007).

Histone displacement is not only linked to DNA replication but also can occur spontaneously or during processes such as transcription (Ahmad and Henikoff, 2002; Rando and Ahmad, 2007; Schwartz and Ahmad, 2005) and DNA repair (reviewed in Soria et al., 2012). However, as synthesis of canonical histones is restricted to the S phase, replication-independent histone variants are used as replacements outside of the S phase. One such example is the H3.3 histone variant that is expressed throughout the cell cycle (Ahmad and Henikoff, 2002). The general localization patterns of H3.3, around the promoters and within the gene bodies of expressed genes, is consistent with the idea that H3.3 is deposited as a replacement histone at transcribed genes (Goldberg et al., 2010; Kraushaar et al., 2013; Schwartz and Ahmad, 2005; Wirbelauer et al., 2005). Furthermore, a number of studies indicated that H3.3 is preferentially marked by modifications associated with active euchromatin and depleted of silencing modifications (Hake et al., 2006; McKittrick et al., 2004), leading to the notion that H3.3 is predominantly associated with transcription and is a marker for active chromatin. However, H3.3 also has been detected at pericentric heterochromatin (PCH) and telomeres, regions of silenced constitutive heterochromatin, and the role of H3.3 at these sites is currently unclear (Drané et al., 2010; Goldberg et al., 2010; Wong et al., 2009, 2010).

Intriguingly, the deposition of H3.3 into these two distinctive chromatin compartments appears to be dependent on two separate pathways. Deposition of H3.3 at euchromatin and bivalent-chromatin promoters is mediated by HIRA, an H3.3-specific histone chaperone (Banaszynski et al., 2013; Goldberg et al., 2010; Ray-Gallet et al., 2002). Concordantly, HIRA shows strong co-localization with H3.3 and active histone modifications across the genome (Goldberg et al., 2010; Kraushaar et al.,

CrossMark

2013; Pchelintsev et al., 2013), and HIRA knockouts (KOs) fail to deposit H3.3 at euchromatic sites in the genome, including promoters, gene bodies, and enhancers (Goldberg et al., 2010; Pchelintsev et al., 2013). In contrast, deposition of H3.3 at PCH and telomeres is dependent on a second complex comprised of Daxx and ATRX (Drané et al., 2010; Goldberg et al., 2010; Wong et al., 2010). Both ATRX and Daxx are required for the deposition of H3.3 at these repeats, and mutations in the ATRX/Daxx/H3.3 pathway have been linked to certain cancers and an alternative lengthening of telomeres (ALT) phenotype (Heaphy et al., 2011; Jiao et al., 2011; Lovejoy et al., 2012; Schwartzentruber et al., 2012), demonstrating the importance of this pathway in maintaining chromatin integrity and, with it, genomic stability.

In this study, we expanded on the role of the ATRX/Daxx complex in H3.3 deposition across the genome. Here we show that ATRX-dependent deposition of H3.3 is not limited to PCH and telomeres, but also occurs at other distinct heterochromatic regions dispersed throughout the genome, most notably at the silenced alleles of imprinted genes. Using these imprinted genes as a model, we demonstrate that ATRX-dependent deposition of H3.3 preferentially localizes to the DNA-methylated allele, indicating that this process must be driven epigenetically. Finally, we demonstrate that H3.3 deposition is crucial for the somatic maintenance of H3K9me3 at many constitutive heterochromatic regions, and we propose a role for this histone variant in maintaining the epigenetic memory of silenced heterochromatin.

RESULTS

ATRX and H3.3 Are Enriched at Heterochromatic Repeats throughout the Genome

Previously published data of validated ATRX chromatin immunoprecipitation sequencing (ChIP-seq) in mouse embryonic stem cells (ESCs) were obtained from the GEO database (GSM551138) (Law et al., 2010), and reads were aligned to genomic repeats using Repeats Enrichment Estimator (Day et al., 2010). Consistent with previous studies, ATRX was enriched at pericentric and telomeric repeats relative to input (Figure 1A; Goldberg et al., 2010; Law et al., 2010). In addition, ATRX was enriched at a number of other genomic repeats (Figure S1). As genomic repeats are known to be enriched for H3K9me3 and H4K20me3, chromatin modifications that are associated with heterochromatin, all ATRX-binding sites across the genome also were tested for association with these two repressive modifications.

ChIP-seq reads for ATRX, H3K9me3 (GSM1375155) (Bulut-Karslioglu et al., 2014), and H4K20me3 (GSM656527) were aligned to the genome with Bowtie (Langmead, 2010), and 1,643 ATRX-binding sites were identified in mouse ESCs. H3K9me3 and H4K20me3 ChIP-seq reads were quantitated under ATRX peaks and compared to random genomic fragments generated with RSA-tools (http://rsat.ulb.ac.be/rsat/random-genome-fragments_form.cgi; Figure 1B). Both H3K9me3 and H4K20me3 were enriched under ATRX-binding sites relative to random control regions, indicating that ATRX is consistently associated with these two heterochromatic modifications throughout the genome. This is also consistent with a recent study showing that the ATRX ADD domain is able to enrich for H3K9me2/3- and H4K20me2/3-modified nucleosomes (Su et al., 2014).

ATRX binding at pericentric and telomeric repeats has been linked to the deposition of the histone variant H3.3 into these regions (Drané et al., 2010; Goldberg et al., 2010; Wong et al., 2010). Analysis of ChIP-seq results for histone H3.3 confirmed that this histone variant was enriched at telomeric and PCH (Figure 1C, left). Given the strong relationship between ATRX and H3.3 at repetitive heterochromatin, all ATRX-binding sites throughout the genome were profiled for H3.3 enrichment. Similar to the findings for H3K9me3 and H4K20me3, ATRX genomic targets were generally enriched for H3.3 relative to random genomic controls (Figure 1C, right). Intriguingly, ATRX-binding sites were found to be simultaneously enriched for the two histone marks of heterochromatin, H3K9me3 and H4K20me3, as well as for H3.3 (Figure 1D).

We next looked for primary DNA sequence features that might underlie ATRX-binding sites and found that ATRX primarily localizes to subsets of tandem repeats (n = 880) and endogenous retroviral (ERVK) repeats (n = 247) in mouse ESCs. In addition, ATRX was found to localize to CpG islands (CGIs) (n = 56) with a higher frequency than expected by chance, though CGI targets represent a relatively minor proportion of total ATRX peaks (Figures 1E and 1F). The majority of ATRX-binding sites in mouse ESCs therefore can be classified as binding either tandem repeats, ERVK repeats, or CGIs, which are enriched for heterochromatic modifications (H3K9me3/H4K20me3) and histone H3.3 (Figure 1F, bottom; Figure S2).

ATRX Localizes to CGIs Associated with an Atypical Chromatin Signature

ATRX-dependent deposition of histone H3.3 at telomeres and PCH has been described previously and the co-localization of H3K9me3 and H4K20me3 is consistent with the heterochromatic nature of these regions. We have now extended this association to other ATRX-binding sites, which, in the main, comprise interstitial repeats that also appear to form segments of heterochromatin.

In contrast, CGIs usually are associated with gene promoters, transcription, and modifications associated with active chromatin, and the association with ATRX is unexplained. We therefore decided to examine chromatin modifications associated with the identified ATRX-bound CGIs in more detail. Only 75 CGIs in the genome are overlapped by ATRX peaks, and in total 56 ATRX peaks that overlie CGIs were identified (some ATRX peaks overlie more than one CGI). Typical of ATRX targets, but unusual for CGIs, ATRX-bound CGIs were simultaneously enriched for H3K9me3, H4K20me3, and histone H3.3 (Figures 2A and 2C). In addition, half of the ATRX-bound CGIs were DNA methylated (GSE28254) (Brinkman et al., 2012), a modification that is present at only 5% of CGIs genome wide (Figure 2B).

Sequential filters applied to CGIs across the genome isolated several factors associated with ATRX binding. Selecting for CGIs enriched for modifications associated with heterochromatin (DNA methylation, H3K9me3, and H4K20me3) increased the proportion of CGIs bound by ATRX (Figure 2D, left). Further selection for CGIs associated with H3.3 enrichment and active

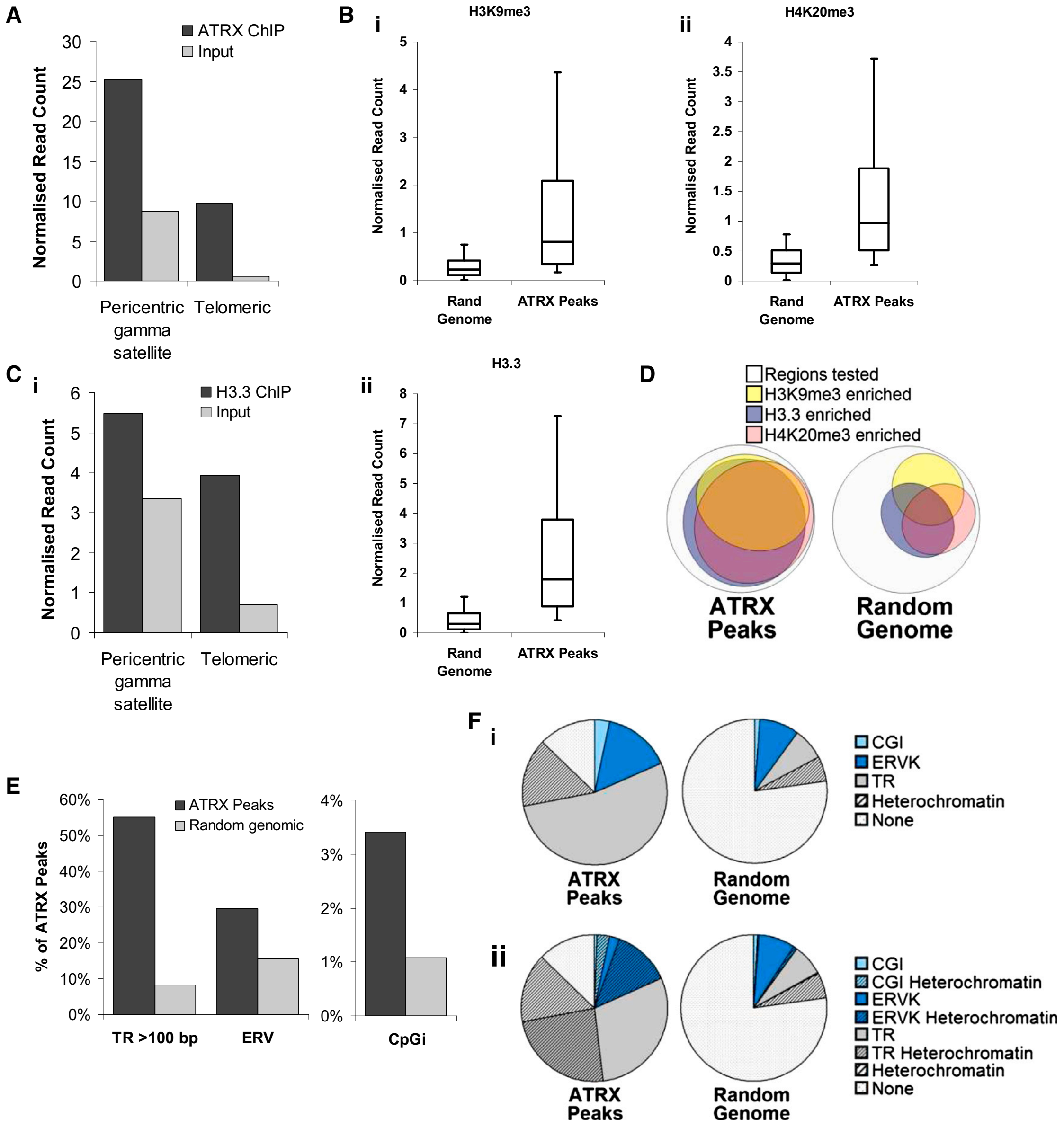

Figure 1. ATRX Localizes to Genomic Regions that Are Enriched for Heterochromatic (H3K9me3/H4K20me3) Modifications and Histone H3.3

(A) ATRX ChIP-seq reads are mapped to genomic repeats and normalized for total read count compared to input.

(B) (Left) H3K9me3 and (right) H4K20me3 are enriched under ATRX peaks compared to random genomic fragments. H3K9me3 and H4K20me3 ChIP-seq reads were quantitated under ATRX peaks and normalized for total read counts. Normalized read counts under random genomic fragments were used as a control. Boxes represent the 25th, median, and 75th percentiles; whiskers represent the 90th and 10th percentiles.

(C) (Left) H3.3 ChIP-seq reads are mapped to genomic repeats and normalized for total read count compared to input. (Right) H3.3 ChIP-seq quantitation under ATRX peaks are normalized for total read count.

(D) Overlap between heterochromatic H3K9me3 and H4K20me3 marks and histone H3.3 under ATRX peaks. Enrichment is defined as normalized read counts >75th percentile of random genomic fragments. Overlap of H3K9me3/H4K20me3/H3.3 enrichments in random fragments of the genome is shown as a control.

(E) ATRX is enriched at tandem repeats (TRs), ERVK elements, and CGIs (CGI) in the genome relative to random genomic fragments.

(F) Proportion of ATRX-binding sites that overlie CGI, TRs, ERVK, and heterochromatin. See also Figure S1.

transcription (determined by RNA sequencing [RNA-seq]) increased the ATRX-bound fraction to 90% (Figure 2D, left). Assessment of ATRX peaks that overlapped CGIs showed that 28 of 56 (50%) of these ATRX peaks were not associated with promoters (Table S1), compared to a genomic average of 23%. The majority (32 of 56) of ATRX-associated CGIs were found to be localized to intragenic regions and embedded within a transcriptional unit (Table S1; Figure 2E), and they

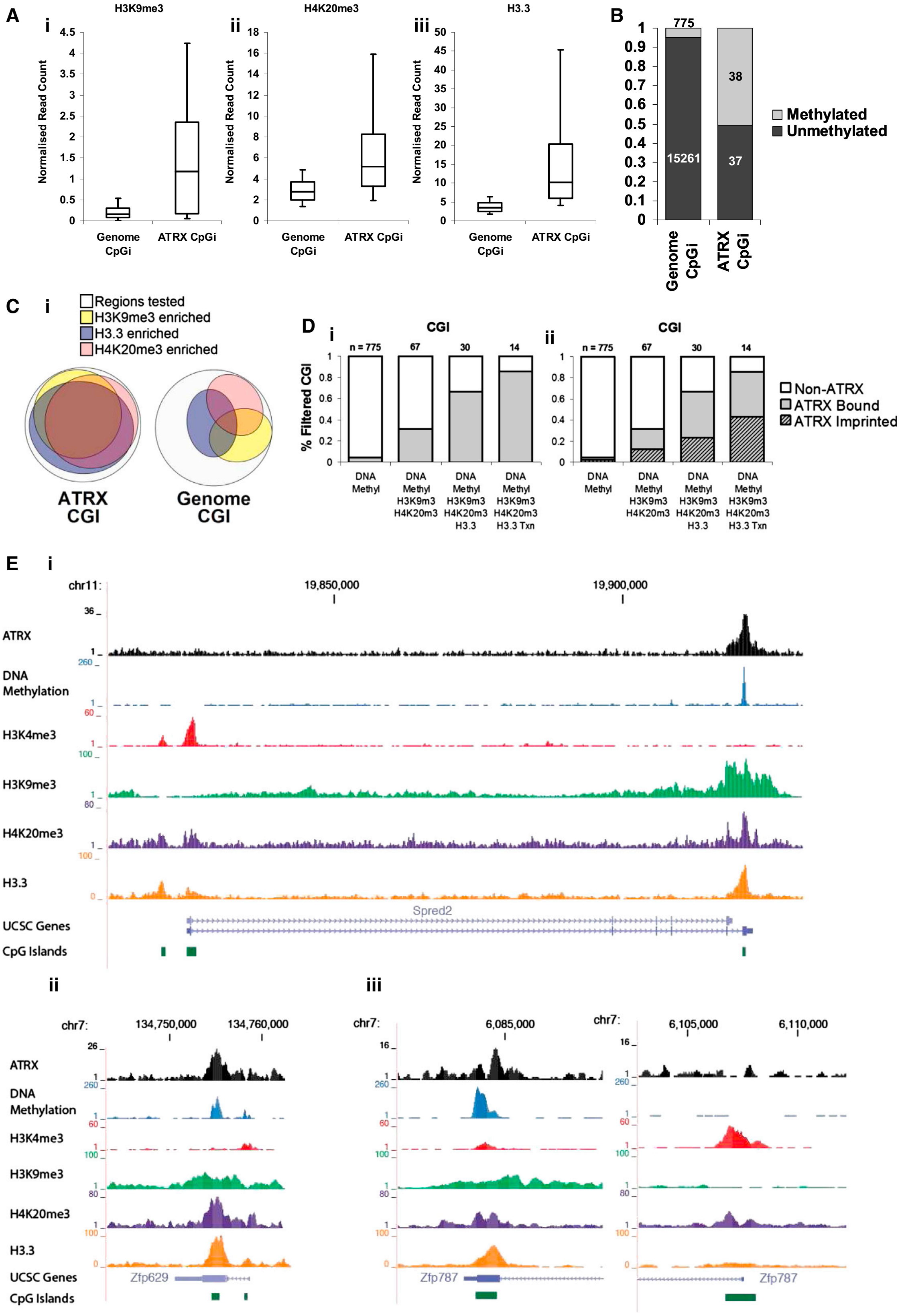

(legend on next page)

demonstrated a different profile of chromatin marks from those seen at promoter CGIs (Figure 2E, top). The ATRX-binding sites that overlapped CGI promoters showed minimal overlap between the peak of ATRX binding and the CGI, indicating these overlaps may represent a distinct subclass (Figures S2D–S2F). ATRX binding therefore is associated with a particular subset of heterochromatin-modified intragenic CGIs, which also are enriched for H3.3 and transcribed.

Imprinted, Differentially Methylated Regions Are Enriched for ATRX, Heterochromatic Modifications, and H3.3

Filtering for features associated with ATRX-bound CGIs also led to an enrichment for imprinted loci (Figure 2D, right; Table S1). Imprinted genes are specialized genes that are expressed in a monoallelic fashion, depending on the parental origin of the allele. There are an estimated 150 imprinted genes in the mouse genome, most of which are arranged in chromosomal clusters (Williamson et al., 2014). The imprinted expression pattern of multiple genes within a cluster can be controlled by single imprinting control region (ICR). These specialized regions, which commonly have the characteristics of CGIs, are marked by DNA methylation on one of the two parental alleles. As this differential DNA methylation originates from one of the two germlines, these regions are referred to as germline differentially methylated regions (DMRs). Enrichment of ATRX was found at 12 of the 17 germline DMR regions that have been identified in the mouse genome thus far (Table 1), and modest ATRX enrichment (although not peak-called) was detected at an additional three germline DMRs (Figure S3). In addition, ATRX binding was detected at four imprinting-associated somatic DMRs, meaning that ATRX peaks were seen at 16 of 21 (80%) of these highly specialized regions of the genome (Table 1); 13 of these were included in the list of ATRX-bound CGIs (Table S1). Interestingly, the two DMRs that lacked any detectable enrichment of ATRX, *Gnas* and *Slc38a4*, also were depleted of other factors (H3K9me3, H4K20me3, and H3.3) associated with ATRX binding. ATRX enrichment at selected imprinted regions was further confirmed by ChIP qPCR in wild-type (WT) versus ATRX KO mouse ESCs (Figure S4A), and examples of these sites are shown (Figure S4B).

ATRX localization at these regions coincided with enrichment for heterochromatic modifications and histone H3.3 (Table 1; Figure 3A, top). All ICRs and DMRs across the genome were associated with DNA methylation, generally high levels of H3K9me3 and H3.3, and mid-to-high levels of H4K20me3 (Table 1), the same chromatin signature linked to ATRX. This means imprinted regions are linked with both ATRX binding and its associated chromatin signature, implying a specialized function for this particular combination of chromatin marks in the genome.

ATRX and Histone H3.3 Preferentially Localize to the Methylated Allele of Imprinted DMRs

Imprinted DMRs in the genome are characterized by differential chromatin modifications, depending on the parental origin of the allele (Fournier et al., 2002; Kelsey and Feil, 2013; Regha et al., 2007; Singh et al., 2011). In general, one parental allele is modified with DNA methylation and histone modifications associated with silenced heterochromatin, while the other allele is modified with marks of active chromatin, such as H3K4me3, associated with promoter activity on this parental allele. Screenshots of selected imprinted DMRs in the mouse genome (Figure 3A, top) illustrate some of the chromatin modifications that are known to be associated with these regions. Importantly, as ChIP-seq does not normally distinguish between alleles, the active (H3K4me3) and heterochromatic (DNA methylation, H3K9me3, and H4K20me3) modifications appear to overlap at these regions, although these modifications are known to be distributed allelically (Fournier et al., 2002; Pannetier et al., 2008; Singh et al., 2011).

Given that ATRX, through its ADD domain, is capable of recognizing H3K9me3 (Dhayalan et al., 2011; Eustermann et al., 2011; Iwase et al., 2011) while H3.3 is widely associated with active chromatin, we wondered if these two proteins also might be allelically distributed. This can be investigated using cells derived from F1 offspring of parental mouse strains that harbor distinctive SNPs, such as an inbred female SV129 crossed to an outbred *Mus m. castaneus* male (SV129 × Cast F1). Publically available ChIP-seq of H3K4me3 (GSM307605) and H3.3 (GSM487542) in mouse ESCs derived from a SV129 × Cast cross were obtained and aligned to the mouse genome. Reads that spanned an informative SNP at relevant imprinted DMRs were extracted and assigned to either the maternal or paternal allele. H3K4me3 reads primarily localized to the unmethylated allele, as previously reported, but, unexpectedly, H3.3 reads were consistently found to be biased toward the opposite, DNA-methylated allele (Figure 3A, bottom).

This finding was further confirmed by performing ChIP against ATRX, H3.3, H3K9me3, and the activating H3K4me3 modifications in mouse ESCs derived from a similar female SV129 × Cast male cross. Resulting ChIP products were amplified with primers designed around polymorphic SNPs, and PCR products were digested with restriction enzymes specific for either the

Figure 2. ATRX-Bound CGI Are Enriched for Marks of Heterochromatin (H3K9me3/H4K20me3 and DNA Methylation) and H3.3

(A) Normalized read counts of (left) H3K9me3, (middle) H4K20me3, and (right) H3.3 ChIP-seq at ATRX-bound CGI relative to genomic CGI are shown. Boxes represent the 25th, median, and 75th percentiles; whiskers represent the 90th and 10th percentiles.

(B) Proportion of CGI that are methylated genome-wide in comparison to ATRX-bound CGI is shown.

(C) Overlapping enrichment profiles of H3K9me3, H4K20me3, and H3.3 under ATRX CGI compared to genomic CGI are shown.

(D) (Left) Proportion of CGI that are bound by ATRX following sequential filtering for DNA methylation, H3K9me3/H4K20me3 enrichment, H3.3 enrichment, and active transcription is shown. (Right) Proportion of CGI that are associated with imprinting and ATRX after sequential filtering is shown.

(E) Screenshots of the (top) *Spred2* locus, (bottom left) *Zfp629* locus, and (bottom right) *Zfp787* locus show a typical promoter CGI marked by high H3K4me3 and mid-level H3.3 compared to an ATRX-bound intragenic CGI, which is enriched with marks of heterochromatin (DNA methylation, H3K9me3, and H4K20me3) and H3.3. See also Figure S2.

Table 1. Germline Somatic DMRs Associated with Genomic Imprinting Are Frequently Bound by ATRX

DMR	Chr	Start	End	Feature	Description	CpGi	Methylated	ATRX	H3.3[c]	Daxx[c]	H3 K9me3[c]	H4 K20me3[c]	H3 K4me3[c]	H3 K27me3[c]
Germline DMRs	chr1	63246988	63247117	Zdbf2	maternal	no[a]	yes	yes	mid	mid	high	low	mid	low
	chr2	174119625	174123318	Nespas-Gnasxl	maternal	yes	yes	yes	mid	high	high	high	low	high
	chr2	174152576	174154820	Gnas	maternal	yes	yes	no	mid	mid	mid	mid	low	high
	chr6	4695857	4699483	Peg10	maternal	yes	yes	mid[b]	high	mid	high	high	mid	mid
	chr6	30684007	30689966	Peg1	maternal	yes	yes	yes	high	high	high	mid	mid	mid
	chr7	6680055	6685400	Peg3	maternal	yes	yes	yes	high	high	high	mid	low	mid
	chr7	67148447	67150182	Snrpn	maternal	no[a]	yes	yes	high	mid	high	low	mid	low
	chr7	149765988	149768095	H19	paternal	no[a]	yes	yes	mid	mid	high	mid	low	mid
	chr7	150479567	150482810	Lit1/Kcnq1ot1	maternal	yes	yes	mid[b]	high	high	high	high	mid	mid
	chr9	89771586	89778464	Rasgrf1	paternal	no[a]	yes	mid[b]	high	mid	mid	low	low	high
	chr10	12809849	12811804	Zac1	maternal	yes	yes	yes	high	high	high	high	mid	high
	chr11	11925463	11927100	Meg1/Grb10	maternal	yes	yes	yes	high	mid	high	high	low	high
	chr11	22871974	22872993	Commd1	maternal	yes	yes	yes	high	high	mid	high	mid	low
	chr12	110765047	110769203	IG-DMR	paternal	yes	yes	yes	high	high	high	high	mid	mid
	chr15	96884878	96885292	Slc38a4	maternal	no[a]	no	no	mid	mid	mid	mid	mid	mid
	chr17	12934169	12935733	Igf2r/Airn	maternal	yes	yes	yes	high	mid	high	mid	low	high
	chr18	13130435	13133135	Impact	maternal	yes	yes	yes	high	high	high	high	high	low
Somatic DMRs	chr2	157385786	157386317	Nnat	maternal	yes	yes	yes	high	high	high	high	low	mid
	chr6	58856717	58857141	Herc3	maternal	yes	yes	yes	high	high	high	high	low	mid
	chr7	135831483	135832096	Inpp5f	maternal	yes	yes	yes	high	high	high	high	mid	mid
	chr15	72639967	72640553	Trappc9	maternal	yes	yes	yes	high	high	high	high	low	mid

ATRX peaks were observed at 11 of 17 germline DMRs in the genome and showed mid-level enrichment at three more DMRs. ATRX also bound to five somatic DMRs in mouse ESCs. All regions were marked by DNA methylation and associated with high levels of H3K9me3 and H3.3. See also Figure S3. Low, <25th percentile of genome CpGi; mid, >25th and <75th percentile of genome CpGi; high >75th percentile of genome CpGi.

[a]Manually defined CpGi based on GC content.

[b]Low-level ATRX enrichment missed in peak calls.

[c]Categorized based on normalized read count relative to genome CpGi.

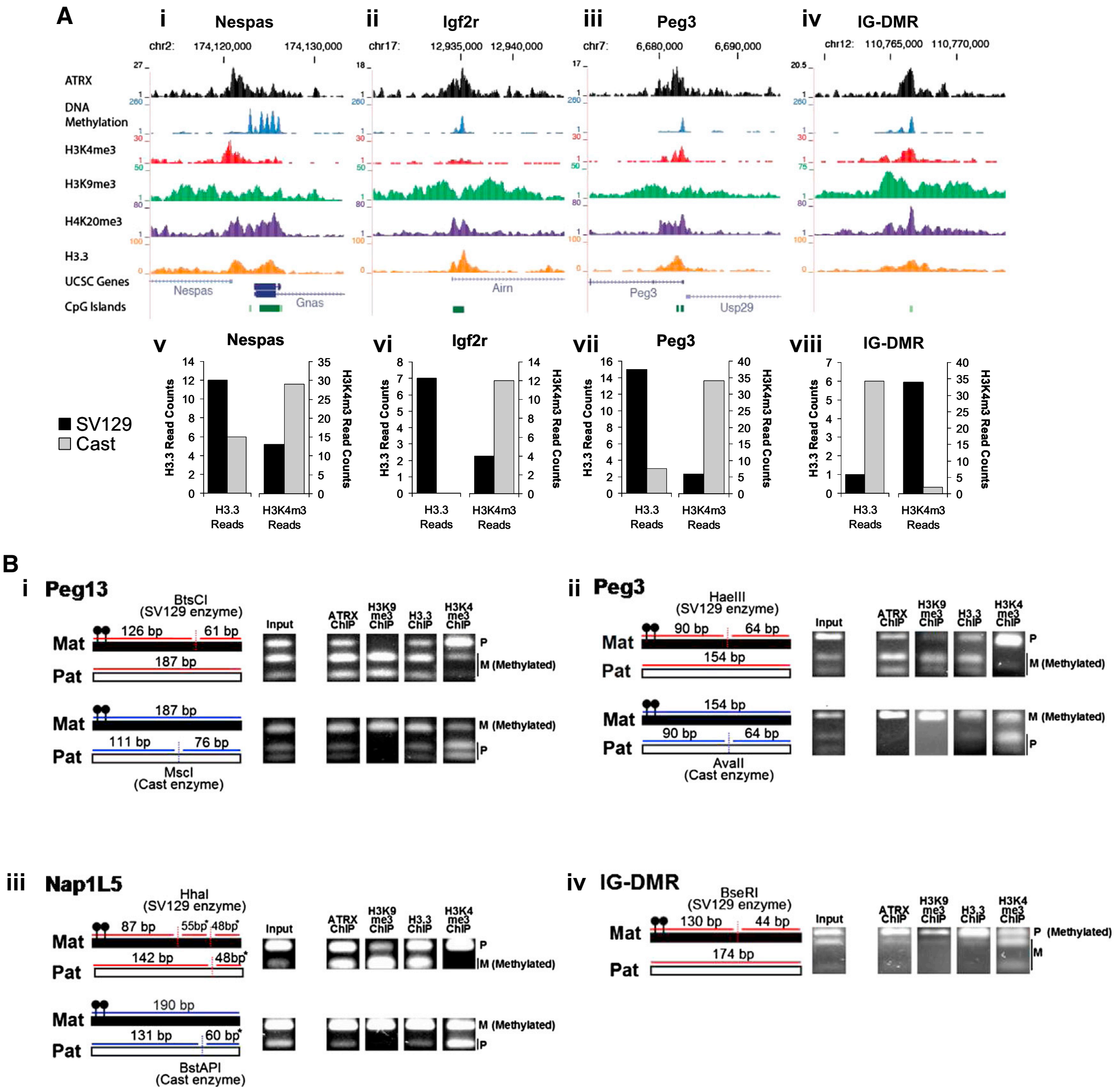

Figure 3. ATRX Localizes Specifically to Methylated DMR in Imprinted Regions along with Histone H3.3 and ATRX and H3.3 Both Localize Specifically to the Methylated Allele

(A) (Top) Screenshots show overlapping chromatin modifications at imprinted DMRs. Imprinted DMRs are simultaneously enriched for H3K4me3-activating marks and heterochromatic marks (DNA methylation, H3K9me3, and H4K20me3). ATRX and H3.3 also are enriched at these regions. (Bottom) Allelic distribution of H3.3 and H3K4me3 is shown. ChIP-seq reads of H3.3 and H3K4me3 in F1 Cast × 129 mouse ESCs with informative SNPs were assigned to either maternal or paternal alleles.

(B) Allele specific localization of ATRX, H3K9me3, H3.3, and H3K4me3 at (top left) *Peg13*, (top right) *Peg3*, (bottom left) *Nap1L5*, and (bottom right) IG-DMR. ChIP products were PCR amplified and digested with allele-specific restriction enzymes. Products were separated by agarose gel electrophoresis. See also Figure S4.

maternal or paternal allele. A number of imprinted loci were tested and digestion patterns were compared to input controls (Figure 3B; Figures S4C–S4F). In agreement with previous studies (Fournier et al., 2002; Singh et al., 2011), the heterochromatic H3K9me3 modification preferentially localized with the reported DNA-methylated allele, while the active H3K4me3 modification localized to the opposite unmethylated allele.

As predicted, ATRX was found to co-localize with the H3K9me3-modified methylated allele, and, intriguingly, H3.3 also was enriched on the DNA-methylated allele. Digestion patterns of H3.3 ChIP PCR products were more similar to H3K9me3 and ATRX than H3K4me3 patterns at all polymorphic imprinted DMRs tested (Figure 3B; Figures S4C–S4F). Therefore, ATRX and H3.3 both localize preferentially with the heterochromatic H3K9me3, DNA-methylated allele at imprinted DMRs.

ATRX Is Required for H3.3 Deposition and H3K9me3 at Imprinted DMRs in the Genome

Given that ATRX is necessary for the correct deposition of H3.3 at PCH and telomeres, we wondered if ATRX also might be necessary for the deposition of H3.3 at imprinted loci. ChIP-seq of H3.3 was performed in WT and ATRX KO mouse ESCs,

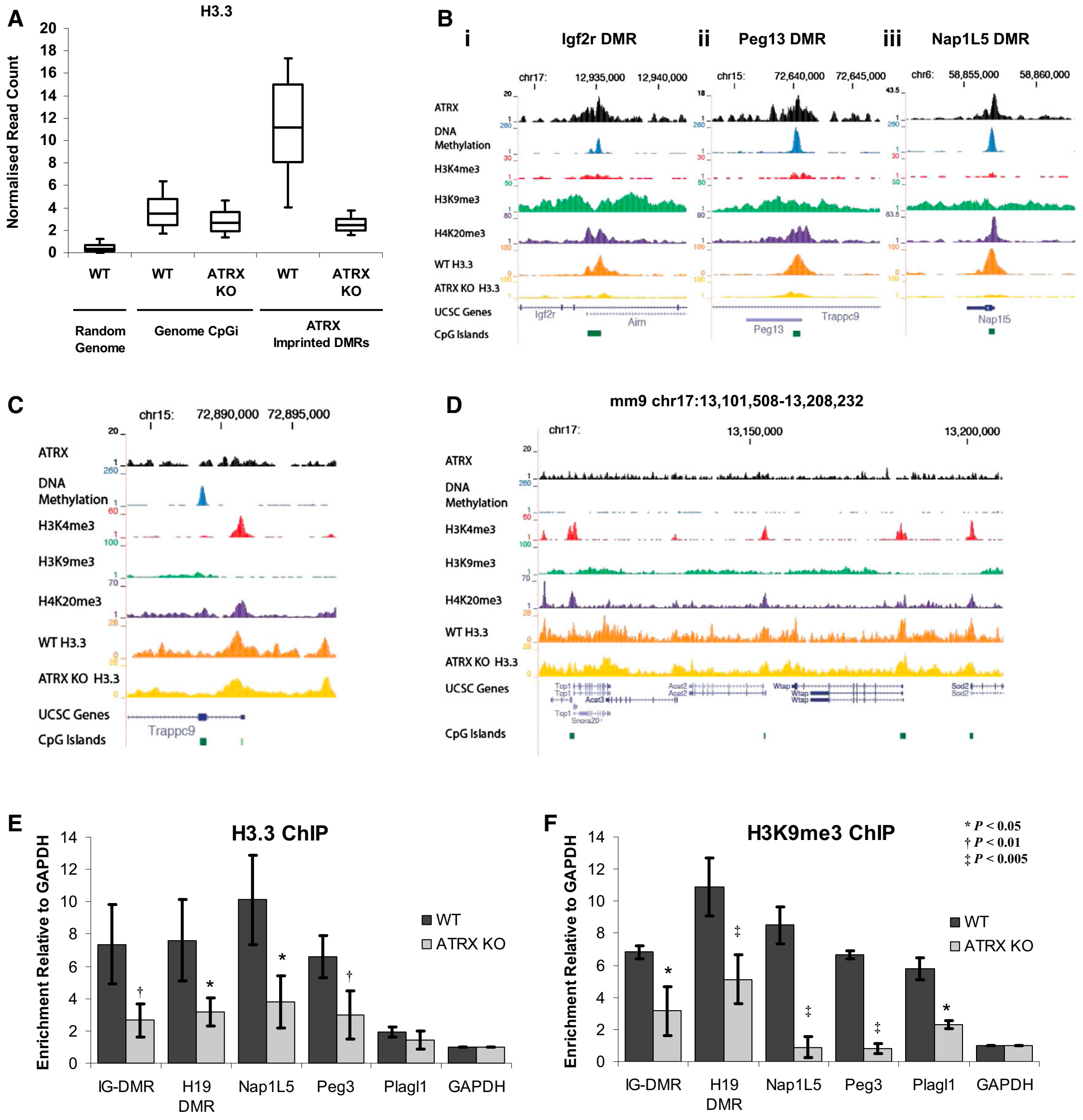

Figure 4. ATRX Is Required for Correct Deposition of H3.3 and H3K9me3 at DMRs

(A) Normalized read counts of H3.3 ChIP-seq in WT versus ATRX KO mouse ESCs at various genomic regions. ATRX-bound imprinted DMRs are highly enriched for H3.3 and enrichment is lost in the absence of ATRX. Boxes represent the 25th, median, and 75th percentiles; whiskers represent the 90th and 10th percentiles.

(B) Screenshots show the (left) *Igf2r*/*Airn*, (middle) *Trappc9*/*Peg13*, and (right) *Nap1L5*/*Herc3* DMRs that are enriched for H3.3 in WT cells and lose H3.3 in ATRX KO cells.

(C) Screenshot shows the *Trappc9* promoter that retains H3.3 in ATRX KO cells.

(D) Screenshot of a genomic region (chr17:13,021,841-13,220,327) shows a number of promoters that are enriched for H3K4me3 and H3.3 in WT cells. H3.3 profiles are unchanged in the ATRX KO cells.

(E and F) Relative enrichment of (E) H3.3 and (F) H3K9me3 at DMRs in WT and ATRX KO mouse ESCs. ChIP products were amplified by qRT-PCR and enrichment was calculated relative to GAPDH after normalizing for input. Results represent the mean and SD of three independent experiments. See also Figure S5.

and ATRX-binding sites were assessed for H3.3 enrichment. Consistent with previous reports, genomic CGIs, which generally are associated with promoters, were found to be moderately enriched for H3.3 compared to random genomic fragments (Figure 4A). However, ATRX-bound imprinted DMRs were found to be strikingly enriched for H3.3 in WT cells, and enrichment was lost in the absence of ATRX (Figure 4A). This profile of H3.3 enrichment in WT cells and subsequent depletion in ATRX KO cells was not restricted to ATRX-associated imprinted DMRs, but appeared to be a general feature associated with ATRX binding throughout the genome (Figures S5A–S5D).

Examples of the chromatin profiles of WT ESCs at a number of imprinted DMRs and the loss of H3.3 in ATRX KO cells are shown in Figure 4B. This unique chromatin profile is limited specifically

to imprinted DMRs, since the canonical promoter CGIs of imprinted genes do not bind ATRX and are unaffected in ATRX KO cells (Figure 4C). Furthermore, promoter-associated CGIs in general, which are marked with H3K4me3 (Figure 4D), retain comparable levels of H3.3 enrichment in ATRX KO cells (Figures 4A and 4D).

ChIP qPCR of H3.3 at a number of imprinted DMRs in WT and ATRX KO cells was used to confirm the loss of H3.3 at these sites in the absence of ATRX (Figure 4E). As these sites also were enriched for H3K9me3 (Figure 4B) and ATRX is known to be associated with this modification, we profiled H3K9me3 in the presence and absence of ATRX. Unexpectedly, we found that loss of ATRX also led to the loss of H3K9me3 at all imprinted DMRs tested (Figure 4F). In addition, we profiled a number of non-imprinted ATRX intragenic CGI targets and found a similar situation where ATRX KO led to the loss of both H3.3 and H3K9me3 enrichment at these sites (Figures S5E and S5F). All non-imprinted CGIs profiled were DNA methylated and modified as heterochromatin and located within the intragenic regions of transcribed genes. Furthermore, analysis of DNA methylation at imprinted DMRs in mouse ESCs also demonstrated an erosion of methylation at these sites in ATRX KO cells (Figure S5G).

Taken together, these data suggest that ATRX-bound intragenic CGIs, including imprinted DMRs, have a unique requirement for ATRX-mediated deposition of H3.3 and that these interactions are necessary for the maintenance of H3K9me3 at these sites.

ATRX/H3.3/H3K9me3 Are Required for the Silencing of Numerous Imprinted Genes

Imprinted genes are characterized by monoallelic expression in a parent-of-origin-dependent manner, and imprinted expression is thought to be dependent on chromatin modifications, particularly DNA methylation, associated with the ICRs and DMRs (Barlow, 2011; Weaver and Bartolomei, 2014). As H3K9me3 is linked to DNA methylation and gene silencing, we sought to determine if loss of ATRX and associated loss of H3.3 and H3K9me3 at these sites would lead to aberrant parental expression of imprinted genes.

To assess this, female SV129 mice carrying a floxed allele of the X chromosome-linked *ATRX* gene (Garrick et al., 2006) were crossed with castaneus males to generate a male (SV129 × Cast) F1 ESC line, which would allow allelic discrimination. The SV129 × Cast $ATRX^{Flox}$ ESCs were transduced with an adenoviral Cre-GFP cassette (AdCre) to generate ATRX KO cells. An adenoviral GFP cassette (AdGFP) was used as a negative control. Cells were sorted for GFP expression and RNA was extracted for sequencing. ATRX KO was confirmed by genotyping PCR (Figure S6A), real-time PCR for ATRX expression (Figure S6B), and RNA-seq data (Figure S6C). RNA was separated into PolyA+ and PolyA− fractions to capture both stable and unstable transcripts, and both fractions were subjected to strand-specific RNA-seq. Reads were aligned to the genome, and reads spanning an informative SNP were extracted and assigned to parental genomes using customized Perl scripts (Table S4). Only genes with more than 50 informative SNP reads were considered for analysis.

A control dataset of 1,500 random genes were assessed for parental skewing (Table S4), and the average parental skew was found to be 50% in the PolyA+ fraction and 54% in the PolyA− fraction toward SV129. The 150 imprinted genes (http://www.har.mrc.ac.uk/research/genomic_imprinting/) were assessed for allelic skewing (Table S4). Within the PolyA− fraction, 11 genes showed >66% skewing toward the expected parental allele and one gene was skewed >60% toward the expected parental allele (Figure 5A). Within the PolyA+ fraction, a total of 12 genes showed >66% skewing toward the expected parental allele, while an additional four genes were skewed >60% toward the expected parental allele (Figure 5B).

Focusing on 18 non-redundant genes that demonstrated allelic biases consistent with the literature, we examined their parental read distribution in the ATRX KO (AdCre) dataset. Five of 18 imprinted genes analyzed demonstrated >10% disruption in allelic distribution patterns in ATRX KO cells (Figure 5A), with particular disruptions occurring at the ATRX-bound methylated allele (Figures S6D–S6G). These results were broadly reproducible across a second independent experiment (Figures S6H and S6I), with the more stable PolyA+ fraction demonstrating a higher degree of reproducibility.

One example of an imprinted locus is illustrated in Figure 5C. At the *Commd1/Zrsr1* locus, the *Commd1* gene is expressed from the maternal allele, and the *Zrsr1* promoter, which is intragenic with respect to *Commd1*, is methylated on the SV129 maternal allele. Data from previous experiments indicate that ATRX, H3.3, and H3K9me3 also are localized to the maternal SV129 allele. On the reciprocal paternal allele, *Commd1* is silent and *Zrsr1* is unmethylated and expressed. RNA-seq data confirmed *Zrsr1* is preferentially expressed from the Castaneus paternal allele (Figure 5B), while the expression from the maternal SV129 allele is suppressed. In ATRX KO cells, there is a substantial increase in *Zrsr1* reads originating from the SV129 maternal allele compared to WT cells (Figure 5C). This suggests that loss of ATRX leads to the loss of H3.3 and H3K9me3 and results in the de-repression of the normally silenced maternal SV129 *Zrsr1* allele.

In total, five genes demonstrated >10% disruption in allelic distributions across four imprinted loci; Commd1/Zrsr1, Nespas, Sgce, and Trappc9 (Figures S6D–S6G). Where appropriate, the reads for the overlapping gene partner also have been illustrated. This indicates that aberrant allelic expression is a general feature associated with the loss of ATRX at these sites (Figures S6D–S6G).

DISCUSSION

Histone H3.3 is a replication-independent histone variant (Ahmad and Henikoff, 2002) that is indispensable in mammalian development (Bush et al., 2013). Numerous studies have indicated that H3.3 primarily is associated with transcription (Goldberg et al., 2010; Kraushaar et al., 2013; Schwartz and Ahmad, 2005; Wirbelauer et al., 2005) and is enriched for modifications associated with euchromatin (Hake et al., 2006; McKittrick et al., 2004). The deposition of H3.3 into these euchromatic regions is dependent on the HIRA chaperone complex (Ray-Gallet et al., 2002), and HIRA KO leads to the loss of H3.3 at these sites

OPEN
ACCESS
CellPress

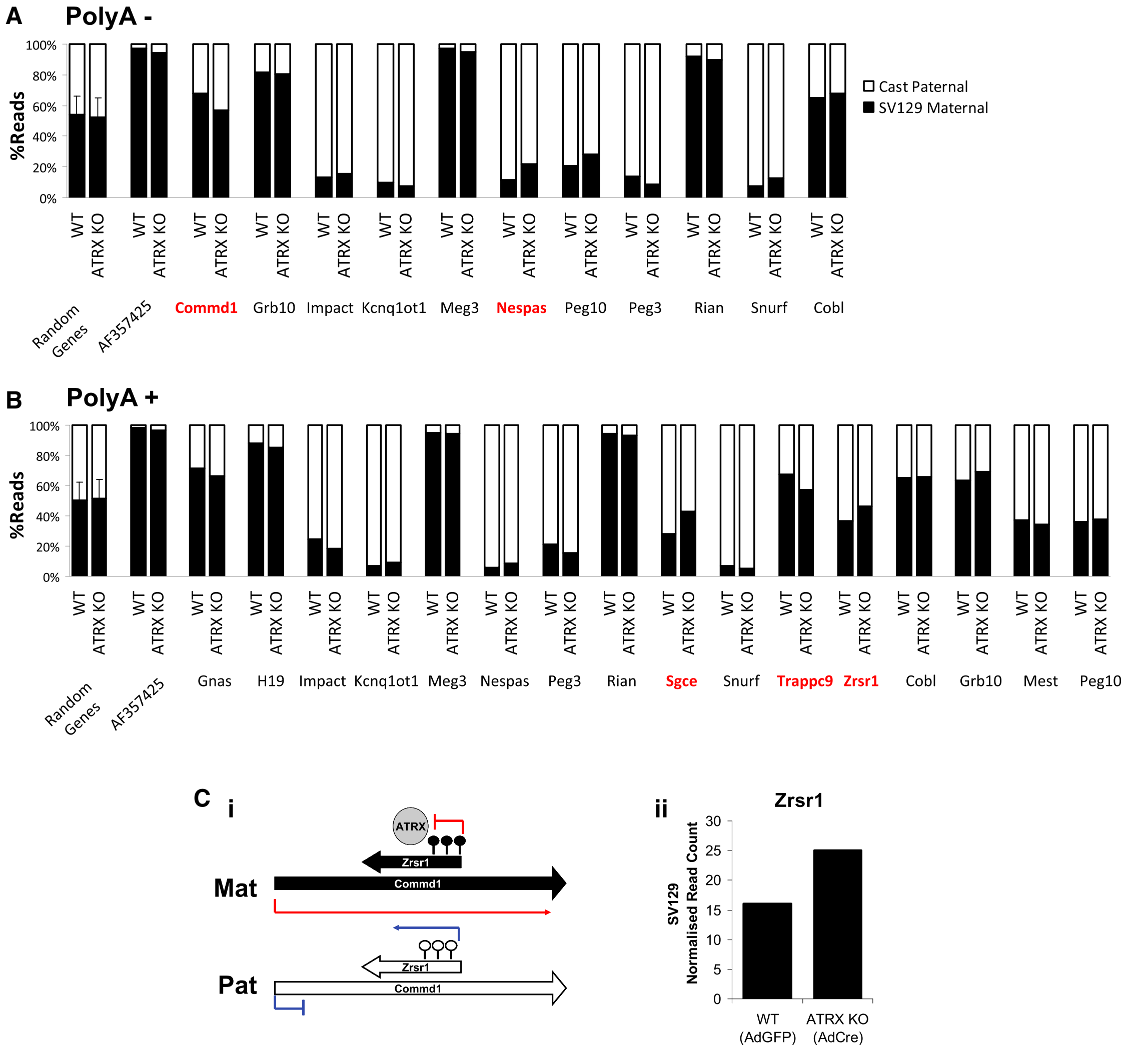

Figure 5. Allelic Read Distribution at Imprinted Genes Is Disrupted in ATRX KO Cells

(A and B) RNA-seq of WT and ATRX KO ESCs show skewed distribution of reads at a number of imprinted genes in the (A) PolyA− and (B) PolyA+ fractions in WT cells. Genes where allelic distribution differed by >10% in ATRX KO compared to WT cells are indicated in red. Allelic distribution of reads across randomly chosen genes in the genome are shown for comparison. Error bars represent SD of allelic skewing across the random dataset.

(C) (Left) A schematic representation of the Zrsr1/Commd1 imprinted locus shows allelic expression and methylation patterns. (Right) Normalized read counts arise from the maternal SV129 Zrsr1 allele in WT and ATRX KO cells. See also Figure S6.

(Goldberg et al., 2010; Pchelintsev et al., 2013). However, histone H3.3 also is associated with a second chaperone complex, ATRX/Daxx, which is required for the deposition of H3.3 into PCH and telomeres, regions that are considered constitutive heterochromatin (Drané et al., 2010; Goldberg et al., 2010; Wong et al., 2010). In this study, we show that this association is not limited to PCH and telomeres, but also applies to distinct heterochromatic regions distributed throughout the genome where the presence or absence of ATRX has important functional consequences on gene silencing.

Genome-wide analysis of ATRX-binding sites showed that these were enriched for both heterochromatic marks (H3K9me3 and H4K20me3) and H3.3. Among these were short tandem repeats and intragenic CGIs, which included many imprinted DMRs. Here we chose to focus on the CGI targets of ATRX. In addition to enrichment for H3K9me3 and H4K20me3, ATRX-bound CGI sequences were also more likely to be methylated than the genomic average. The overwhelming majority of annotated CGIs in the genome localized to gene promoters and were enriched for either H3K4me3 or H3K27me3 in a manner that reflects gene expression. In contrast, ATRX-bound CGIs tended to localize within gene bodies, and the atypical chromatin profile observed at these sites likely could be attributed to this genomic position. However, as these heterochromatic CGIs lie within active genes, these regions are nonetheless transcribed, and our data suggest that transcription per se is a factor that positively selects for ATRX binding. As H3.3 is known to act as a replacement histone (reviewed in Elsaesser et al.,

2010), this link to transcription and the associated nucleosomal turnover could partially explain the presence of H3.3 within these intragenic regions.

H3.3 is generally considered a mark of active chromatin, and, consistent with this, we found co-localization of H3.3 with H3K4me3-modified promoters within our dataset. The deposition of H3.3 into gene-rich regions is thought to be predominantly dependent on HIRA (Pchelintsev et al., 2013; Ray-Gallet et al., 2002) rather than the ATRX/Daxx complex, a finding that is also generally consistent with our data. We found that H3.3 was moderately enriched at CGI promoters across the genome and this enrichment was unaffected in ATRX KO cells, confirming that H3.3 deposition at these regions was generally independent of ATRX. However, the ATRX-bound CGIs identified in this study represent distinct exceptions to this general pattern. At these particular heterochromatin-modified regions, ATRX binding coincided with unusually high enrichment for H3.3 and deposition of H3.3 failed to occur in the absence of ATRX. Interestingly, the CGIs that were positive for all the features associated with ATRX binding also included a substantial number of imprinted DMRs.

ATRX previously has been reported as being involved in silencing at imprinted loci, but in these reports ATRX was required for developmental silencing of the active allele in post-natal brain (Kernohan et al., 2010, 2014). Quite separate from this phenomenon, we showed here that, in mouse ESCs, ATRX acts to maintain the differential expression of the alleles at imprinted loci. We found that at the majority of DMRs in mouse ESCs, the silenced alleles were enriched for marks of constitutive heterochromatin, ATRX, and histone H3.3. The imprinted DMRs represent a unique and functionally distinct set of genomic targets. At these developmentally essential regions, though the two parental alleles are genetically identical, they carry distinct combinations of chromatin modifications (Kelsey and Feil, 2013; Regha et al., 2007; Singh et al., 2011; reviewed in Weaver and Bartolomei, 2014). Thus, imprinted DMRs represent an excellent in vivo model system for assessing chromatin-driven ATRX binding. Allelic discrimination assays at these sites showed that both ATRX and H3.3 were preferentially localized to the DNA-methylated/H3K9me3-modified allele, rather than the H3K4me3-modified allele, at many of the imprinted DMRs. This indicates that epigenetic factors play an important role in targeting ATRX-dependent deposition of H3.3 at imprinted loci in this cellular context.

We previously noted that ATRX-bound CGIs were localized to intragenic regions rather than promoters. However, at imprinted DMRs, these features were not mutually exclusive. A number of imprinted genes were arranged in overlapping transcription pairs, whereby the promoter of one gene was intragenic with respect to a second transcriptional unit. In fact, this pattern of overlapping antisense-oriented transcription is thought to be important for the establishment of imprinting at many loci (reviewed in Barlow, 2011). Using the *Commd1/Zrsr1* locus as an example, the maternally methylated DMR is intragenic with respect to *Commd1* transcription, but, on the unmethylated paternal allele, the same CGI acts as a promoter for *Zrsr1* expression. Therefore, it is likely that the heterochromatin islet at the maternal *Commd1* intragenic CGI suppresses expression of *Zrsr1* from this allele. However, the process of transcription through *Commd1* is predicted to disrupt the heterochromatin-modified nucleosomes at this intragenic CGI, and, in the absence of active maintenance, could potentially lead to the erosion of modifications at this site over time. Results from this study indicate that ATRX-dependent deposition of H3.3 plays a key role in maintaining the heterochromatic modifications at these sites, as loss of ATRX leads to the loss of both H3.3 and H3K9me3 at imprinted DMRs and results in aberrant allelic expression of a number of imprinted genes. Although we observed only modest changes in gene expression in our acute KO model of ATRX, H3.3 KO has been shown to result in changes in expression of imprinted genes (Banaszynski et al., 2013; Bush et al., 2013). ATRX KO in SV129 × Cast ESCs was lethal and, therefore, it was not possible to assess these effects in long-term culture or mouse models. Nonetheless, these data identify ATRX as a regulator of genomic imprinting in this ESC model.

The preferential localization of ATRX and H3.3 to the DNA-methylated allele of many imprinted DMRs indicates that the chromatin environment is an important factor in determining whether ATRX is recruited for the deposition of H3.3. A similar situation was observed at several non-imprinted CGIs located at intragenic regions of actively transcribed genes. These intragenic CGIs were also DNA methylated and marked as heterochromatin, and localization of ATRX to these sites further supports the idea of chromatin-driven binding. Finally, we showed a decrease in both H3.3 and H3K9me3 at these sites in the absence of ATRX, which, in turn, led to aberrant allelic expression. This finding may have broader implications for ATRX-dependent deposition of H3.3 at other genomic sites, such as telomeres, where loss of ATRX and H3.3 also may lead to the loss of heterochromatic modifications.

We hypothesize that ATRX localizes to distinct heterochromatic regions when these sites are subjected to nucleosomal disruptions and that ATRX/Daxx helps to facilitate the timely deposition of H3.3. The presumption is that ATRX/Daxx then leads to the recruitment of other factors, including histone lysine methyltransferase(s) such as Eset/Setdb1/Kmt1e, which controls the H3K9me3 at imprinted DMRs (Girardot et al., 2014; Yuan et al., 2009) that can modify the newly deposited replacement histones, thus ensuring the faithful somatic maintenance of silencing modifications (Figures 6A–6C). In the absence of ATRX, H3.3 is no longer deposited at its target loci. This is associated with the loss of H3K9me3, de-repression, and aberrant transcription of the genes involved (Figure 6D). The recruitment of ATRX to these sites is most likely driven by the combinatorial interaction of ATRX with both H3K9me3 and HP1 (Eustermann et al., 2011; Iwase et al., 2011) and transcription. At the heterochromatic allele of imprinted DMRs, the Eset-mediated H3K9me3 is recognized by HP1γ (Pannetier et al., 2008), and our recent studies suggest Eset can interact with ATRX, at least in vitro (data not shown). This would provide a reinforcing feedback loop in which the ATRX-mediated incorporation of H3.3 and its ensuing methylation of lysine-9 facilitate the recruitment of ATRX to the heterochromatic regions involved.

OPEN ACCESS
CellPress

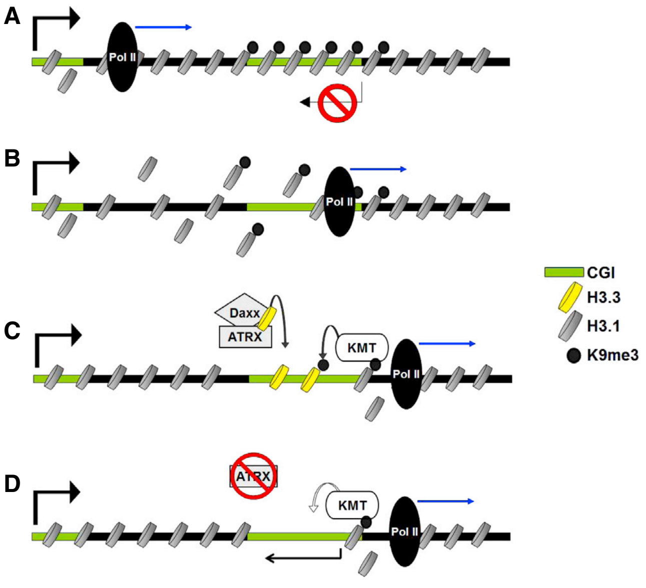

Figure 6. ATRX-Mediated Deposition of H3.3 Maintains Heterochromatic Profiles during Transcription

(A) Schematic depiction of a gene containing a gene body CGI. Heterochromatic modifications such as H3K9me3 silence the internal CGI to prevent cryptic transcription.

(B) Progression of RNA Pol II during transcription disrupts heterochromatin-modified nucleosomes at the internal CGI.

(C) ATRX/Daxx mediate deposition of replacement H3.3 at the heterochromatic CGI and facilitate maintenance of chromatin modifications at this site.

(D) Loss of ATRX leads to failure of H3.3 deposition, loss of chromatin modifications, and aberrant transcription.

EXPERIMENTAL PROCEDURES

GEO Datasets

A list of GEO datasets used in this study can be found in Table S5.

ChIP

ATRX and Daxx ChIP were performed as previously described (Law et al., 2010). In brief, cells were fixed in PBS with 2 mM EGS (Pierce 26103) for 45 min at room temperature followed by 1% formaldehyde for 20 min. Chromatin was sonicated to <500 bp and lysates were immunoprecipitated with 10 μg ATRX H300 (Insight Biotechnology sc-15408), 10 μg Daxx M-112 (Santa Cruz Biotechnology sc-7152), or rabbit IgG control (Dako X0903). Histone ChIPs were performed according to the manufacturer's instructions (Millipore 17-295) using either 10 μg H3.3 antibody (Millipore 09-838), 5 μg H3K9me3 antibody (Abcam ab8898), or 2 μg H3K4me3 antibody (Abcam ab8580).

ChIP-Seq Analysis

Fastq files were aligned to the mouse (mm9) genome with Bowtie (v1.0.0) (Langmead, 2010) using default settings, except that reads were allowed to match the genome up to five times to facilitate mapping to repeats. ATRX peak calls were performed in Seqmonk (v0.24.1) (http://www.bioinformatics.babraham.ac.uk/projects/seqmonk/), and reads associated with histone modifications were quantitated under ATRX peaks after normalizing for total mapped reads. Further details are provided in the Supplemental Experimental Procedures.

Repeats Analysis

Fastq files were aligned to genomic repeats using Repeat Enrichment Estimator v1.0. Mapped reads were normalized for total read count and compared to input samples. Repeats with less than 50 mapped reads were excluded from further analysis. Repeat classes were considered enriched for protein binding if ChIP-seq reads exceeded input reads by more than 2-fold after normalizing for total read counts.

Identification of SNPs in ChIP-Seq Reads

ChIP-seq data of H3K4me3 (GSM307605) and H3.3 (GSM487542) in female SV129 × Castaneus male F1 ESCs were obtained from GEO. SNPs that differed between these two strains were obtained from The Mouse Genomes Project at the Wellcome Trust Sanger Institute webpage (https://www.sanger.ac.uk/resources/mouse/genomes/). ChIP-seq reads were mapped using Bowtie and reads that overlapped DMRs were extracted. As the IG-DMR was significantly broader than the ATRX-binding site, only reads underlying the ATRX peak were extracted from this region. DMR reads were overlapped with SNPs and parental SNPs were identified using custom Perl scripts (available upon request).

Allelic Discrimination Assays

ATRX-bound DMRs were assessed for differential SNPs that created a differential restriction site. Primers were designed around appropriate sites and used to amplify ChIP DNA. PCRs were performed using Expand High-Fidelity Taq (Roche 11732641001) according to the manufacturers' instructions. ChIP or input DNA (1 μl) was amplified in a 50-μl reaction for 40 cycles with an annealing temperature of 55°C. Equal amounts of PCR products were subjected to restriction digest and separated on a 2.5% agarose gel. Digestion products were visualized using Sybr Gold (Life Technologies S-11494). Digested PCR products were quantified in ImageJ and allelic enrichments were calculated relative to input. A list of primers used in this study can be found in Table S2.

Real-Time qPCR

Real-time qPCR was performed using SYBR green mastermix (Applied Biosystems 4309155). ChIP enrichments were determined relative to a three-point dilution series of input DNA and normalized relative to GAPDH enrichment. A list of primers used in this study can be found in Table S3.

Generation of SV129 × Castaneus ATRX KO ESCs

SV129 females carrying an ATRX^{Flox} cassette (Garrick et al., 2006) were mated with Castaneus males and ESCs were derived from male ATRX^{Flox} embryos. An adenoviral-packaged Cre-recombinase cassette with a GFP marker (Vector Laboratories 1779) (AdCre) was used to generate ATRX KO ESCs. An adenoviral-packaged eGFP cassette (AdGFP) was used as a negative control (Vector Laboratories 1768). One million ESCs were seeded in a T25 flask and transduced with 1×10^8 plaque-forming units (PFU) of either AdCre or AdGFP adenovirus in 10 ml media. Transduced cells were recovered for 48 hr and then subjected to fluorescence-activated cell sorting (FACS) for GFP expression. AdCre GFP-positive cells were confirmed as ATRX KO by genotyping PCR and qPCR for ATRX expression. AdGFP-positive cells were retained as a negative control.

RNA Extraction and Sequencing

RNA was extracted at 5 days post-transduction with Tri-Reagent (Sigma-Aldrich T9424). Total RNA was separated using a PolyATtract mRNA Isolation System (Promega Z5310). The rRNA was removed from PolyA− fractions with a Ribo-Zero Magnetic Kit (EpiCentre MRZH11124). Libraries were created using NEBNext Ultra Directional RNA Library Prep Kit for Illumina (New England Biolabs E7420L) with minor modifications (Lamble et al., 2013), and sequenced on a HiSeq2500 in rapid mode to generate 50 bp paired-end reads.

Allelic RNA-Seq Analyses

RNA-seq data were aligned to the mm9 genome with TopHat v2.0.10 (Trapnell et al., 2009) using default parameters. Parental SNPs across genes were identified as previously described for ChIP-seq datasets, except that reads were analyzed in a strand-specific manner. RNA-seq reads also were extracted from 1,500 random genes and used to assess the distribution of parental SNPs. Only genes with more than 50 informative SNP reads were included in the analysis.

ACCESSION NUMBERS

The ChIP-seq and RNA-seq datasets generated in this study have been deposited to the NCBI GEO and are available under accession number GSE59271.

SUPPLEMENTAL INFORMATION

Supplemental Information includes Supplemental Experimental Procedures, six figures, and five tables and can be found with this article online at http://dx.doi.org/10.1016/j.celrep.2015.03.036.

AUTHOR CONTRIBUTIONS

H.P.J.V. designed and carried out the experiments. H.P.J.V. and J.R.H. analyzed the data and wrote the paper. C.R. derived the SV129 × Cast ATRX^{Flox} ESCs. I.A.D.L.R.-V. and T.J. provided the H3K9me3 data. R.F. provided reagents, advised on the interpretation of the data, and helped write the paper. D.R.H. and R.J.G. designed the experiments, advised on the interpretation of the data, and wrote the paper.

ACKNOWLEDGMENTS

This work was supported by the Medical Research Council (UK). We thank the High-Throughput Genomics Group at the Wellcome Trust Centre for Human Genetics (funded by Wellcome Trust grant reference 090532/Z/09/Z and MRC Hub grant G0900747 91070) for the generation of the sequencing data. We wish to acknowledge the Computational Biology Research Group, Weatherall Institute of Molecular Medicine, Oxford, for use of their services in this project.

Received: August 8, 2014
Revised: January 30, 2015
Accepted: March 14, 2015
Published: April 9, 2015

REFERENCES

Abmayr, S.M., and Workman, J.L. (2012). Holding on through DNA replication: histone modification or modifier? Cell *150*, 875–877.

Ahmad, K., and Henikoff, S. (2002). The histone variant H3.3 marks active chromatin by replication-independent nucleosome assembly. Mol. Cell *9*, 1191–1200.

Banaszynski, L.A., Wen, D., Dewell, S., Whitcomb, S.J., Lin, M., Diaz, N., Elsässer, S.J., Chapgier, A., Goldberg, A.D., Canaani, E., et al. (2013). Hira-dependent histone H3.3 deposition facilitates PRC2 recruitment at developmental loci in ES cells. Cell *155*, 107–120.

Barlow, D.P. (2011). Genomic imprinting: a mammalian epigenetic discovery model. Annu. Rev. Genet. *45*, 379–403.

Brinkman, A.B., Gu, H., Bartels, S.J., Zhang, Y., Matarese, F., Simmer, F., Marks, H., Bock, C., Gnirke, A., Meissner, A., and Stunnenberg, H.G. (2012). Sequential ChIP-bisulfite sequencing enables direct genome-scale investigation of chromatin and DNA methylation cross-talk. Genome Res. *22*, 1128–1138.

Bulut-Karslioglu, A., De La Rosa-Velázquez, I.A., Ramirez, F., Barenboim, M., Onishi-Seebacher, M., Arand, J., Galán, C., Winter, G.E., Engist, B., Gerle, B., et al. (2014). Suv39h-dependent H3K9me3 marks intact retrotransposons and silences LINE elements in mouse embryonic stem cells. Mol. Cell *55*, 277–290.

Bush, K.M., Yuen, B.T., Barrilleaux, B.L., Riggs, J.W., O'Geen, H., Cotterman, R.F., and Knoepfler, P.S. (2013). Endogenous mammalian histone H3.3 exhibits chromatin-related functions during development. Epigenetics Chromatin *6*, 7.

Day, D.S., Luquette, L.J., Park, P.J., and Kharchenko, P.V. (2010). Estimating enrichment of repetitive elements from high-throughput sequence data. Genome Biol. *11*, R69.

Dhayalan, A., Tamas, R., Bock, I., Tattermusch, A., Dimitrova, E., Kudithipudi, S., Ragozin, S., and Jeltsch, A. (2011). The ATRX-ADD domain binds to H3 tail peptides and reads the combined methylation state of K4 and K9. Hum. Mol. Genet. *20*, 2195–2203.

Drané, P., Ouararhni, K., Depaux, A., Shuaib, M., and Hamiche, A. (2010). The death-associated protein DAXX is a novel histone chaperone involved in the replication-independent deposition of H3.3. Genes Dev. *24*, 1253–1265.

Elsaesser, S.J., Goldberg, A.D., and Allis, C.D. (2010). New functions for an old variant: no substitute for histone H3.3. Curr. Opin. Genet. Dev. *20*, 110–117.

Eustermann, S., Yang, J.C., Law, M.J., Amos, R., Chapman, L.M., Jelinska, C., Garrick, D., Clynes, D., Gibbons, R.J., Rhodes, D., et al. (2011). Combinatorial readout of histone H3 modifications specifies localization of ATRX to heterochromatin. Nat. Struct. Mol. Biol. *18*, 777–782.

Fournier, C., Goto, Y., Ballestar, E., Delaval, K., Hever, A.M., Esteller, M., and Feil, R. (2002). Allele-specific histone lysine methylation marks regulatory regions at imprinted mouse genes. EMBO J. *21*, 6560–6570.

Garrick, D., Sharpe, J.A., Arkell, R., Dobbie, L., Smith, A.J., Wood, W.G., Higgs, D.R., and Gibbons, R.J. (2006). Loss of Atrx affects trophoblast development and the pattern of X-inactivation in extraembryonic tissues. PLoS Genet. *2*, e58.

Girardot, M., Hirasawa, R., Kacem, S., Fritsch, L., Pontis, J., Kota, S.K., Filipponi, D., Fabbrizio, E., Sardet, C., Lohmann, F., et al. (2014). PRMT5-mediated histone H4 arginine-3 symmetrical dimethylation marks chromatin at G + C-rich regions of the mouse genome. Nucleic Acids Res. *42*, 235–248.

Goldberg, A.D., Banaszynski, L.A., Noh, K.M., Lewis, P.W., Elsaesser, S.J., Stadler, S., Dewell, S., Law, M., Guo, X., Li, X., et al. (2010). Distinct factors control histone variant H3.3 localization at specific genomic regions. Cell *140*, 678–691.

Groth, A., Rocha, W., Verreault, A., and Almouzni, G. (2007). Chromatin challenges during DNA replication and repair. Cell *128*, 721–733.

Hake, S.B., Garcia, B.A., Duncan, E.M., Kauer, M., Dellaire, G., Shabanowitz, J., Bazett-Jones, D.P., Allis, C.D., and Hunt, D.F. (2006). Expression patterns and post-translational modifications associated with mammalian histone H3 variants. J. Biol. Chem. *281*, 559–568.

Heaphy, C.M., de Wilde, R.F., Jiao, Y., Klein, A.P., Edil, B.H., Shi, C., Bettegowda, C., Rodriguez, F.J., Eberhart, C.G., Hebbar, S., et al. (2011). Altered telomeres in tumors with ATRX and DAXX mutations. Science *333*, 425.

Iwase, S., Xiang, B., Ghosh, S., Ren, T., Lewis, P.W., Cochrane, J.C., Allis, C.D., Picketts, D.J., Patel, D.J., Li, H., and Shi, Y. (2011). ATRX ADD domain links an atypical histone methylation recognition mechanism to human mental-retardation syndrome. Nat. Struct. Mol. Biol. *18*, 769–776.

Jackson, V. (1988). Deposition of newly synthesized histones: hybrid nucleosomes are not tandemly arranged on daughter DNA strands. Biochemistry *27*, 2109–2120.

Jasencakova, Z., Scharf, A.N., Ask, K., Corpet, A., Imhof, A., Almouzni, G., and Groth, A. (2010). Replication stress interferes with histone recycling and predeposition marking of new histones. Mol. Cell *37*, 736–743.

Jiao, Y., Shi, C., Edil, B.H., de Wilde, R.F., Klimstra, D.S., Maitra, A., Schulick, R.D., Tang, L.H., Wolfgang, C.L., Choti, M.A., et al. (2011). DAXX/ATRX, MEN1, and mTOR pathway genes are frequently altered in pancreatic neuroendocrine tumors. Science *331*, 1199–1203.

Kelsey, G., and Feil, R. (2013). New insights into establishment and maintenance of DNA methylation imprints in mammals. Philos. Trans. R. Soc. Lond. B Biol. Sci. *368*, 20110336.

Kernohan, K.D., Jiang, Y., Tremblay, D.C., Bonvissuto, A.C., Eubanks, J.H., Mann, M.R., and Bérubé, N.G. (2010). ATRX partners with cohesin and MeCP2 and contributes to developmental silencing of imprinted genes in the brain. Dev. Cell *18*, 191–202.

Kernohan, K.D., Vernimmen, D., Gloor, G.B., and Bérubé, N.G. (2014). Analysis of neonatal brain lacking ATRX or MeCP2 reveals changes in nucleosome density, CTCF binding and chromatin looping. Nucleic Acids Res. *42*, 8356–8368.

Kraushaar, D.C., Jin, W., Maunakea, A., Abraham, B., Ha, M., and Zhao, K. (2013). Genome-wide incorporation dynamics reveal distinct categories of turnover for the histone variant H3.3. Genome Biol. *14*, R121.

Lamble, S., Batty, E., Attar, M., Buck, D., Bowden, R., Lunter, G., Crook, D., El-Fahmawi, B., and Piazza, P. (2013). Improved workflows for high throughput library preparation using the transposome-based Nextera system. BMC Biotechnol. *13*, 104.

Langmead, B. (2010). Aligning short sequencing reads with Bowtie. Curr. Protoc. Bioinformatics *Chapter 11*, 7.

Law, M.J., Lower, K.M., Voon, H.P., Hughes, J.R., Garrick, D., Viprakasit, V., Mitson, M., De Gobbi, M., Marra, M., Morris, A., et al. (2010). ATR-X syndrome protein targets tandem repeats and influences allele-specific expression in a size-dependent manner. Cell *143*, 367–378.

Lovejoy, C.A., Li, W., Reisenweber, S., Thongthip, S., Bruno, J., de Lange, T., De, S., Petrini, J.H., Sung, P.A., Jasin, M., et al.; ALT Starr Cancer Consortium (2012). Loss of ATRX, genome instability, and an altered DNA damage response are hallmarks of the alternative lengthening of telomeres pathway. PLoS Genet. *8*, e1002772.

McKittrick, E., Gafken, P.R., Ahmad, K., and Henikoff, S. (2004). Histone H3.3 is enriched in covalent modifications associated with active chromatin. Proc. Natl. Acad. Sci. USA *101*, 1525–1530.

Pannetier, M., Julien, E., Schotta, G., Tardat, M., Sardet, C., Jenuwein, T., and Feil, R. (2008). PR-SET7 and SUV4-20H regulate H4 lysine-20 methylation at imprinting control regions in the mouse. EMBO Rep. *9*, 998–1005.

Pchelintsev, N.A., McBryan, T., Rai, T.S., van Tuyn, J., Ray-Gallet, D., Almouzni, G., and Adams, P.D. (2013). Placing the HIRA histone chaperone complex in the chromatin landscape. Cell Rep. *3*, 1012–1019.

Rando, O.J., and Ahmad, K. (2007). Rules and regulation in the primary structure of chromatin. Curr. Opin. Cell Biol. *19*, 250–256.

Ray-Gallet, D., Quivy, J.P., Scamps, C., Martini, E.M., Lipinski, M., and Almouzni, G. (2002). HIRA is critical for a nucleosome assembly pathway independent of DNA synthesis. Mol. Cell *9*, 1091–1100.

Regha, K., Sloane, M.A., Huang, R., Pauler, F.M., Warczok, K.E., Melikant, B., Radolf, M., Martens, J.H., Schotta, G., Jenuwein, T., and Barlow, D.P. (2007). Active and repressive chromatin are interspersed without spreading in an imprinted gene cluster in the mammalian genome. Mol. Cell *27*, 353–366.

Schwartz, B.E., and Ahmad, K. (2005). Transcriptional activation triggers deposition and removal of the histone variant H3.3. Genes Dev. *19*, 804–814.

Schwartzentruber, J., Korshunov, A., Liu, X.Y., Jones, D.T., Pfaff, E., Jacob, K., Sturm, D., Fontebasso, A.M., Quang, D.A., Tönjes, M., et al. (2012). Driver mutations in histone H3.3 and chromatin remodelling genes in paediatric glioblastoma. Nature *482*, 226–231.

Singh, P., Wu, X., Lee, D.H., Li, A.X., Rauch, T.A., Pfeifer, G.P., Mann, J.R., and Szabó, P.E. (2011). Chromosome-wide analysis of parental allele-specific chromatin and DNA methylation. Mol. Cell. Biol. *31*, 1757–1770.

Soria, G., Polo, S.E., and Almouzni, G. (2012). Prime, repair, restore: the active role of chromatin in the DNA damage response. Mol. Cell *46*, 722–734.

Su, Z., Boersma, M.D., Lee, J.H., Oliver, S.S., Liu, S., Garcia, B.A., and Denu, J.M. (2014). ChIP-less analysis of chromatin states. Epigenetics & chromatin *7*, 7.

Trapnell, C., Pachter, L., and Salzberg, S.L. (2009). TopHat: discovering splice junctions with RNA-Seq. Bioinformatics *25*, 1105–1111.

Weaver, J.R., and Bartolomei, M.S. (2014). Chromatin regulators of genomic imprinting. Biochim. Biophys. Acta *1839*, 169–177.

Williamson, C.M., Blake, A., Thomas, S., Beechey, C.V., Hancock, J., Cattanach, B.M., and Peters, J. (2014). Mouse Imprinting Data and References (Oxfordshire: MRC Harwell). http://www.har.mrc.ac.uk/research/genomic_imprinting/.

Wirbelauer, C., Bell, O., and Schübeler, D. (2005). Variant histone H3.3 is deposited at sites of nucleosomal displacement throughout transcribed genes while active histone modifications show a promoter-proximal bias. Genes Dev. *19*, 1761–1766.

Wong, L.H., Ren, H., Williams, E., McGhie, J., Ahn, S., Sim, M., Tam, A., Earle, E., Anderson, M.A., Mann, J., and Choo, K.H. (2009). Histone H3.3 incorporation provides a unique and functionally essential telomeric chromatin in embryonic stem cells. Genome Res. *19*, 404–414.

Wong, L.H., McGhie, J.D., Sim, M., Anderson, M.A., Ahn, S., Hannan, R.D., George, A.J., Morgan, K.A., Mann, J.R., and Choo, K.H. (2010). ATRX interacts with H3.3 in maintaining telomere structural integrity in pluripotent embryonic stem cells. Genome Res. *20*, 351–360.

Yuan, P., Han, J., Guo, G., Orlov, Y.L., Huss, M., Loh, Y.H., Yaw, L.P., Robson, P., Lim, B., and Ng, H.H. (2009). Eset partners with Oct4 to restrict extraembryonic trophoblast lineage potential in embryonic stem cells. Genes Dev. *23*, 2507–2520.

Cell Reports
Supplemental Information

ATRX Plays a Key Role in Maintaining Silencing at Interstitial Heterochromatic Loci and Imprinted Genes

Hsiao P.J. Voon, Jim R. Hughes, Christina Rode, Inti A. De La Rosa-Velázquez, Thomas Jenuwein, Robert Feil, Douglas R. Higgs, and Richard J. Gibbons

Figure S1

Related to Fig. 1

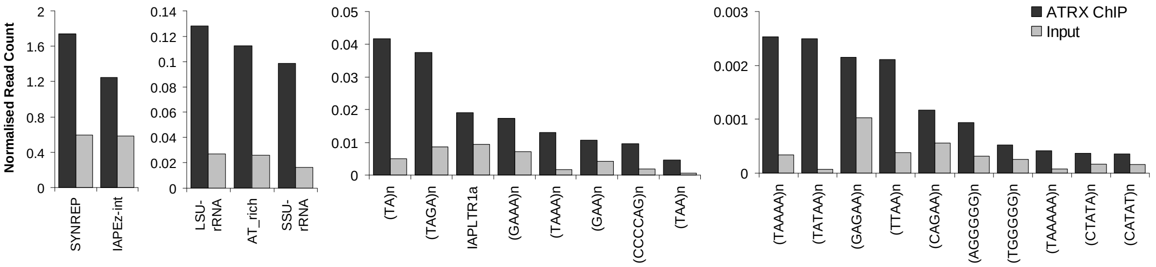

Figure S2

Related to Fig. 2

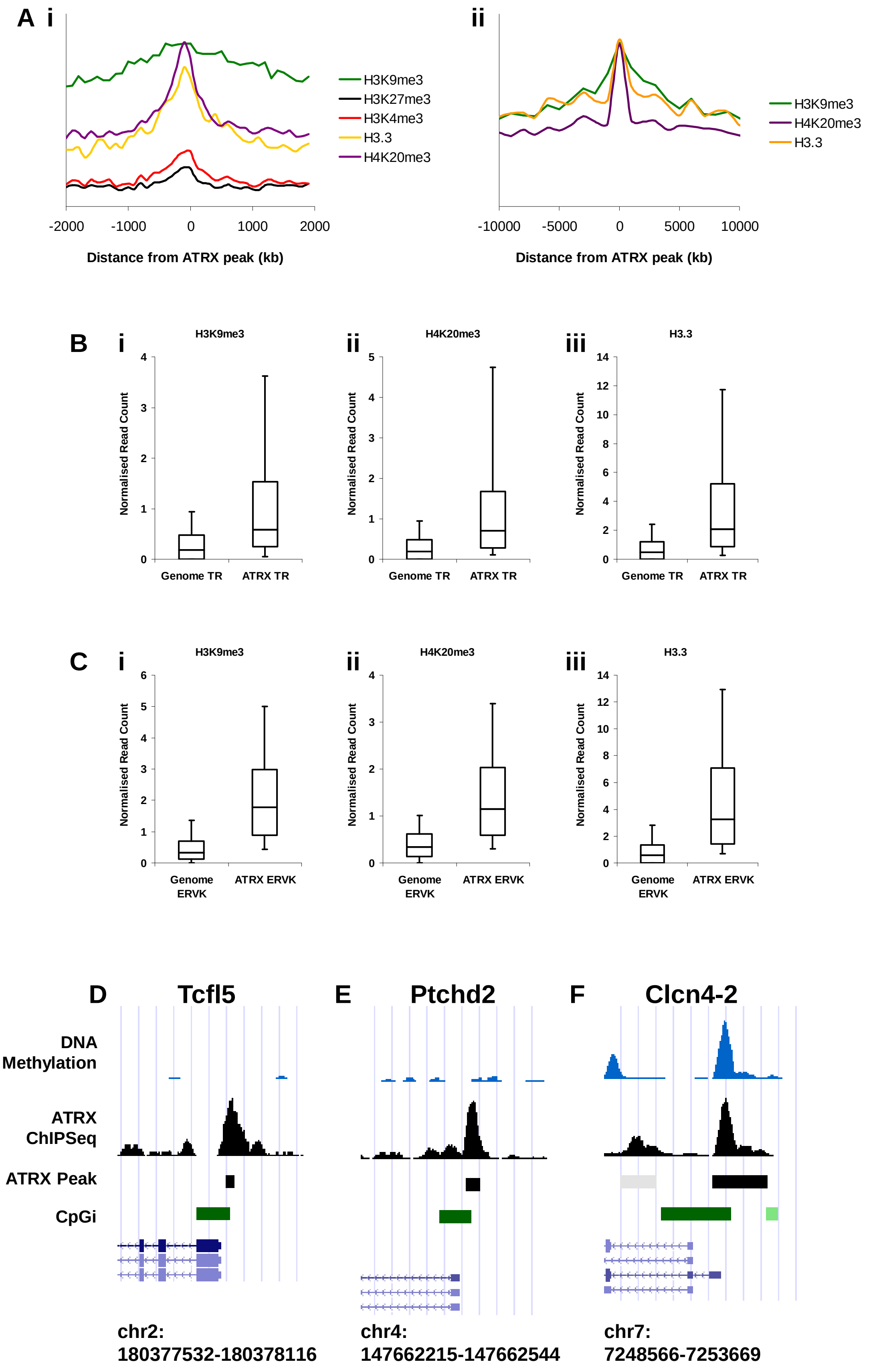

Figure S3

Related to Table 1

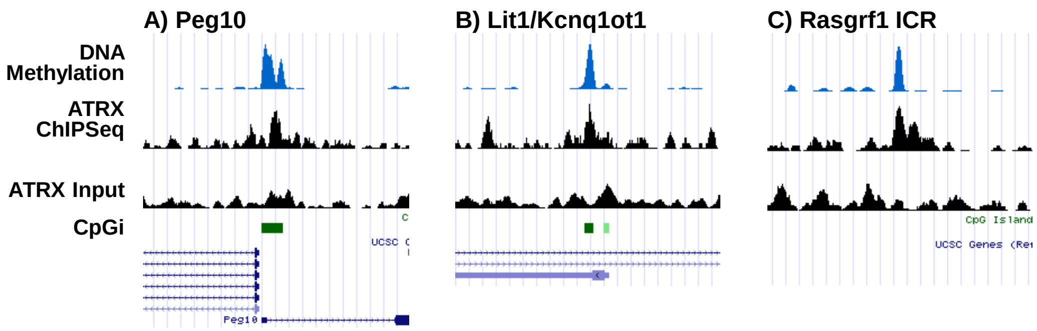

Figure S4

Related to Fig. 3

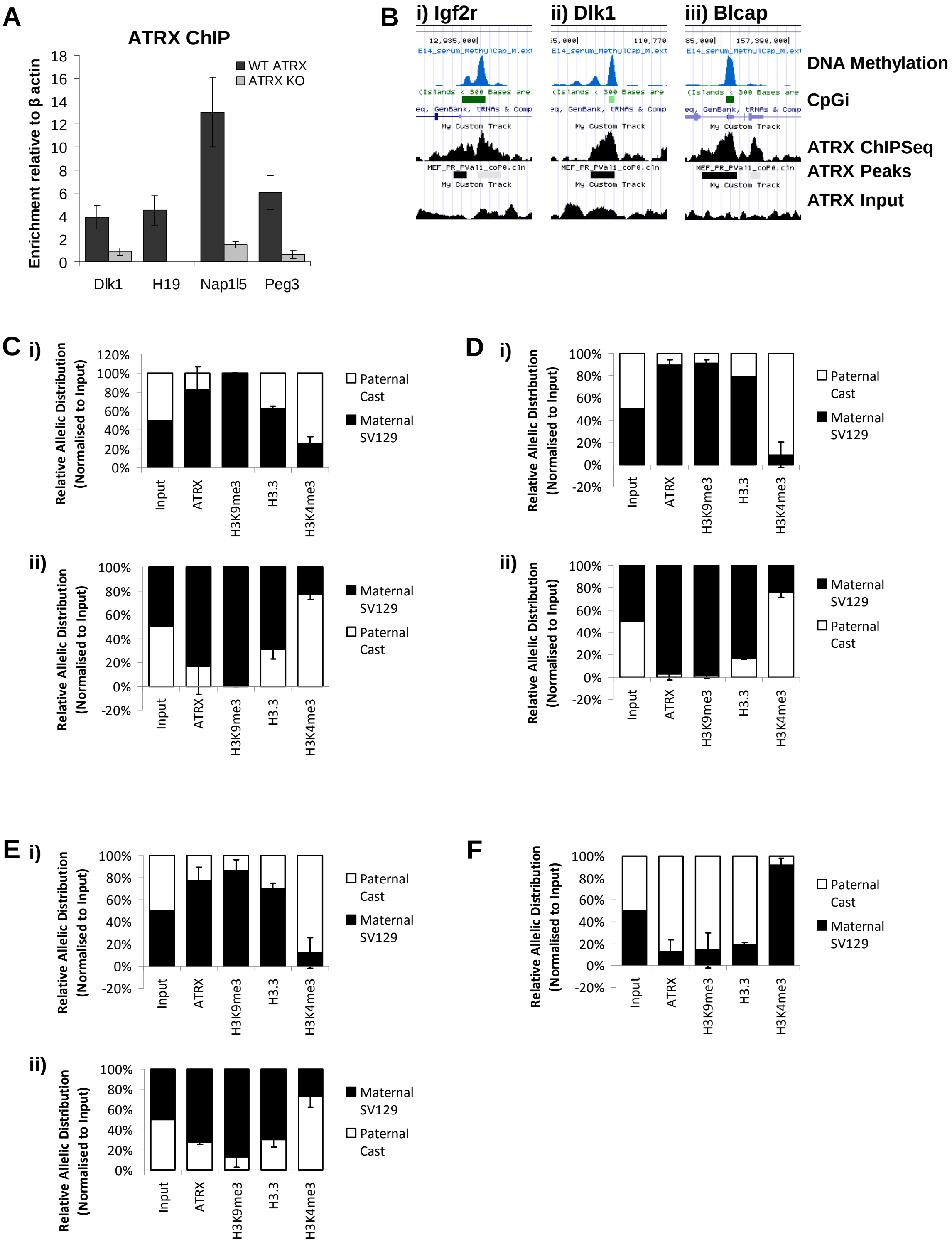

Figure S5

Related to Fig. 4

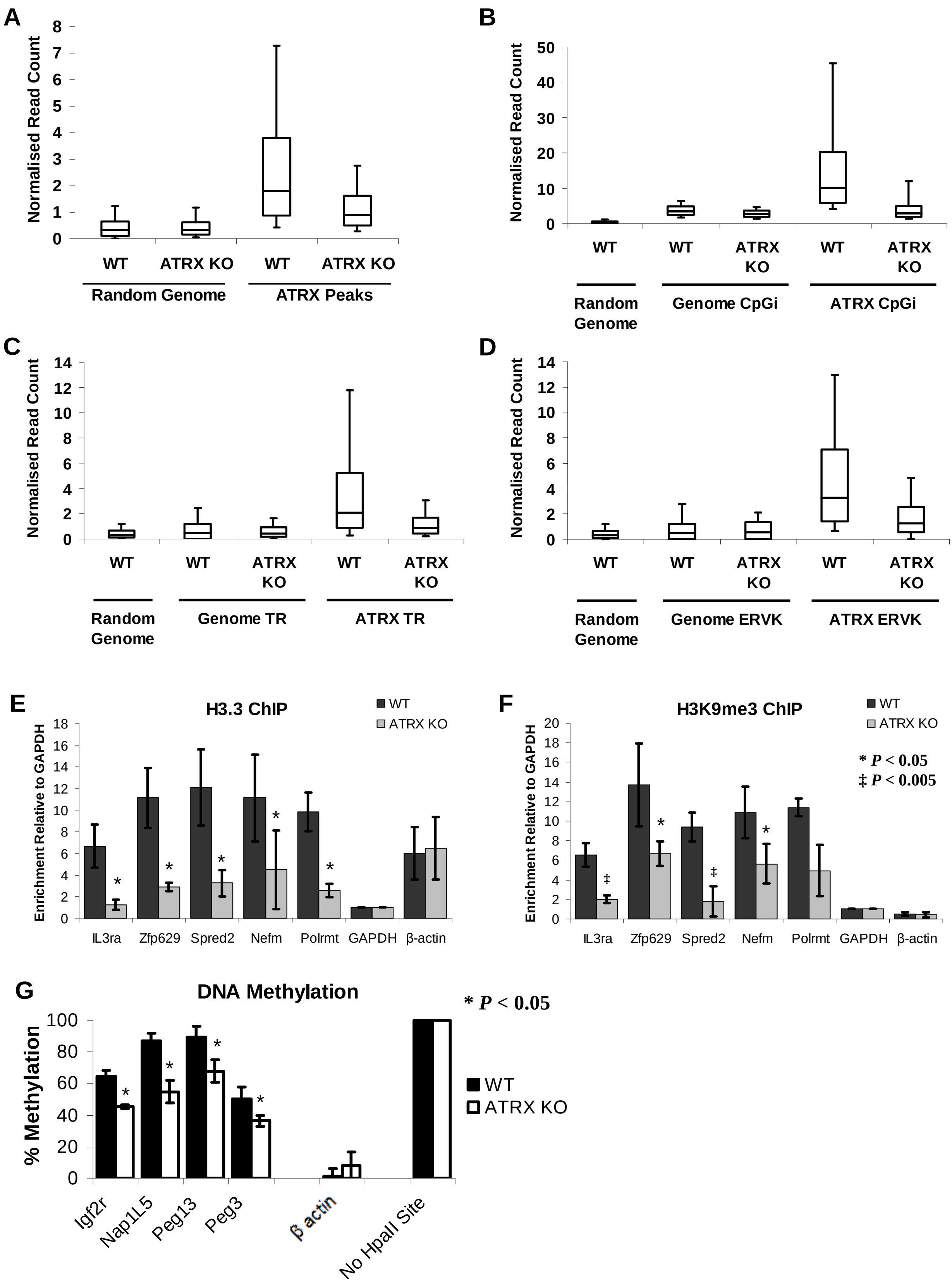

Figure S6

Related to Fig. 5

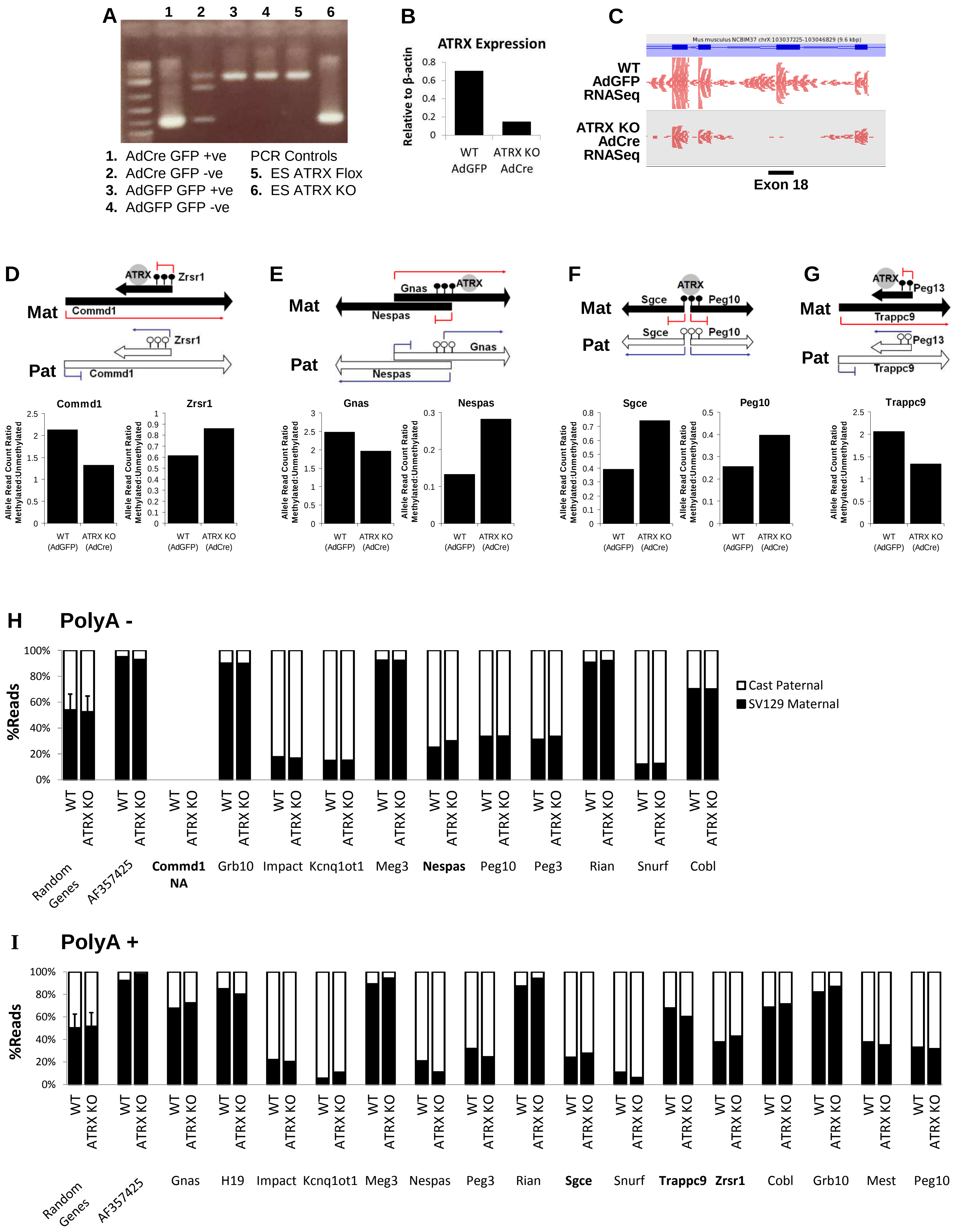

Figure S1, Related to Figure 1. ATRX is enriched at a number of repeats throughout the genome. ATRX ChIP-Seq reads were aligned against genomic repeats and compared against input enrichment after normalising for total read counts.

Figure S2, Related to Figure 2. All ATRX genomic targets are enriched for H3K9me3, H4K20me3 and H3.3. A) Pileup of various chromatin modifications in a **i)** 2 kb and **ii)** 10 kb window around ATRX peaks. ATRX bound regions are highly enriched for H3K9me3, H4K20me3 and H3.3. H3K9me3 enrichment is extended across a broader domain as seen in the 10 kb genomic window. **B)** Average normalised read counts of **i)** H3K9me3 **ii)** H4K20me3 and **iii)** H3.3 at ATRX bound tandem repeats (TR) compared to genomic TRs. **C)** Average normalised read counts of **i)** H3K9me3 **ii)** H4K20me3 and **iii)** H3.3 at ATRX bound ERVK repeats compared to genomic ERVK repeats. Boxes represent the 25th, median and 75th percentile. Whiskers represent the 90th and 10th percentile. ATRX bound TRs and ERVK repeats are enriched for H3K9me3, H4K20me3 and H3.3 compared to respective genomic controls. **D-F)** Examples of ATRX binding at promoter associated CGIs. ATRX binding profiles at **D)** *Tcfl5* **E)** *Ptchd2* and **F)** *Clcn4-2*. Regions where ATRX binding is annotated at promoter associated CGIs tend to show minimal overlap between ATRX peaks and CGIs.

Figure S3, Related to Table 1. Enrichment of ATRX at imprinted DMRs which are missed in peak calls. ATRX ChIP-seq profiles at the **A)** *Peg10* **B)** *Lit1/Kcnq1ot1* and **C)** *Rasgrf1* DMRs. ATRX input sequencing is shown for comparison. ChIP-seq and input tracks are shown on the same scale after normalising for total read counts.

Figure S4, Related to Figure 3. ATRX binds specifically to imprinted DMRs. A) ChIP-qPCR of ATRX at imprinted CpGi. ATRX ChIP was performed in ATRX KO cells as a negative control. Results represent the mean ± standard deviation of three independent experiments. **B)** Screenshots of ATRX ChIP-seq showing localisation specifically at methylated CGI in imprinted genes. **i)** Igf2r DMR **ii)** IG-DMR and **iii)** Blcap/Nnat DMR. **C-F)** Gel quantitation of allelic discrimination ChIP-PCR digests relative to input. Data represent the average of three independent experiments ± standard deviation. **C)** Peg13 digested with **i)** BtsCI **ii)** MscI. **D)** Peg3 digested with **i)** HaeIII **ii)** AvaII. **E)** Nap1L5 digested with **i)** HhaI **ii)** BstAPI and **F)** IG-DMR digested with BseRI.

Figure S5, Related to Figure 4. H3.3 enrichment is a general feature associated with ATRX binding and H3.3 enrichment is lost in ATRX KO cells. A) Average normalised read counts of H3.3 in WT vs ATRX KO cells at all ATRX binding sites across the genome compared to random genomic fragments. **B-D)** Average normalised read counts of H3.3 at major ATRX associated elements in the genome. The genomic average for **B)** CGIs, **C)** TRs and **D)** ERVKs were compared to respective ATRX bound elements in WT vs ATRX KO cells. All sites associated with ATRX enrichment show decreased H3.3 incorporation in ATRX KO cells compared WT cells. Boxes represent the 25th, median and 75th percentile. Whiskers represent the 90th and 10th percentile. ChIP qRT-PCR of **E)** H3.3 and **F)** H3K9me3 at ATRX bound intragenic CGI targets in WT and ATRX KO cells. Enrichment of both H3.3 and H3K9me3 at intragenic CGIs were lost in ATRX KO cells. Results represent the mean ± standard deviations of three independent experiments. **G)** DNA methylation at imprinted DMRs in WT and ATRX KO mouse ES cells. Results represent the mean ± standard deviations of three independent experiments (refer to supplemental methods).

Figure S6, Related to Figure 5. ATRX KO results in aberrant allelic expression of imprinted genes. A) Genotyping PCR of ATRX in GFP sorted cells transduced with AdGFP (WT) or AdCre (ATRX KO). **B)** qRT-PCR of ATRX expression in GFP sorted cells treated with AdGFP and AdCre. **C)** RNA-seq of WT AdGFP and ATRX KO AdCre cells. AdCre treated cells lack reads from exon 18. **D-G)** Ratio of methylated to unmethylated allelic reads from parental SNP informative reads at various imprinted loci. **D)** *Commd1/Zrsr1* **E)** *Gnas/Nespas* **F)** *Sgce/Peg10* and **G)** *Trappc9*. ATRX preferentially localises to the methylated maternal SV129 allele at all depicted loci. ATRX KO cells show disrupted allelic expression profiles compared to WT cells with SV129 maternal reads particularly affected. **H-I)** Differential allelic expression is reproducible in ATRX KO cells. An independent ATRX KO and RNA-seq experiment recapitulated aberrant allelic expression.

Table S1

Related to Fig. 2

Chr	Start	Stop	Promoter Status	Intragenic	Features
1	7386900	7388449	NonPromoter	Intragenic	
1	172993642	173014931	NonPromoter		
4	82158228	82159756	NonPromoter		
4	145424873	145426579	NonPromoter	Intragenic	
4	145723014	145724994	NonPromoter	Intragenic	
4	146559543	146561700	NonPromoter	Intragenic	
5	109977131	109988748	NonPromoter	Intragenic	
6	47693350	47694403	NonPromoter	Intragenic	
6	47727980	47729405	NonPromoter	Intragenic	
6	47971605	47978261	NonPromoter	Intragenic	
6	48543851	48548651	NonPromoter	Intragenic	
6	89784985	89785282	NonPromoter		
7	6081239	6086119	NonPromoter	Intragenic	
7	30577514	30579496	NonPromoter	Intragenic	
7	134750400	134759058	NonPromoter	Intragenic	
10	79195390	79204608	NonPromoter	Intragenic	
10	126949758	126950361	NonPromoter		
11	3023001	3024570	NonPromoter		
11	19917860	19924586	NonPromoter	Intragenic	
12	3234602	3241076	NonPromoter	Intragenic	
12	110764205	110768003	NonPromoter		Imprinted
13	47105262	47107083	NonPromoter	Intragenic	
13	120276362	120284312	NonPromoter	Intragenic	
14	15177661	15188428	NonPromoter	Intragenic	
14	52054268	52056141	NonPromoter	Intragenic	
19	5825902	5848706	NonPromoter		
19	61299571	61305080	NonPromoter	Intragenic	
X	166423462	166437580	NonPromoter	Intragenic	
2	157376955	157393095	Promoter	Intragenic	Imprinted
2	174120474	174124467	Promoter	Intragenic	Imprinted
6	30684845	30692843	Promoter		Imprinted
6	58854617	58859716	Promoter	Intragenic	Imprinted
7	6681284	6683762	Promoter	Intragenic	Imprinted
7	7230112	7232063	Promoter		Monoallelic
7	135828654	135835498	Promoter	Intragenic	Imprinted
10	12805933	12815343	Promoter	Intragenic	Imprinted
11	11918389	11930357	Promoter	Intragenic	Imprinted
11	22870617	22874230	Promoter	Intragenic	Imprinted
15	72639173	72641357	Promoter	Intragenic	Imprinted
17	12932143	12938087	Promoter	Intragenic	Imprinted
18	13130402	13133304	Promoter	Intragenic	Imprinted
17	39979942	39985774	rDNA		Monoallelic
1	90170106	90176974	Promoter		
2	180377532	180378116	Promoter		
3	21975167	21975472	Promoter		
4	120526901	120527621	Promoter		
4	147662215	147662544	Promoter		
5	124560770	124563038	Promoter		
7	7248566	7253669	Promoter		
8	4677024	4680280	Promoter		
8	19782933	19787537	Promoter		
8	19891139	19893565	Promoter		
10	80644767	80645797	Promoter		
12	18139974	18142499	Promoter		
14	8623565	8624031	Promoter		
15	88906698	88906897	Promoter		

Table S2

Related to Fig. 3

		Size	Enzyme	SV129	Cast
Nap1L5_allCh_fwd	TCACCTGTCAGCTTGCTGTGCT	190 bp	HhaI	87, 55, 48 bp	142, 48 bp
Nap1L5_allCh_rev	GCCCCTGCTCGCCAAGATCC		BstAPI	190 bp	131, 60 bp
Peg13_allCh_fwd	GCGGCAGTGTCGCAGGTCTT	187 bp	BtsCI	126, 61 bp	187 bp
Peg13_allCh_rev	CCGAAGAGGACCCGGTGGCT		MscI	187 bp	111, 76 bp
Peg3_allch_fwd	GCCGCTCCGTGGTGATTCCC	154 bp	AvaII	154 bp	90, 64 bp
Peg3_allch_rev2	GCATGCACCCTCTTAGATACCGTCT		HaeIII	90, 64 bp	154 bp
Dlk1_allch_fwd2	GCCGTTCGCTATGAACTACCGCT	174 bp	BseRI	130, 44 bp	174 bp
Dlk1_allch_rev2	GTTCGCGGCACGCGTACACA				

Table S3

Related to Fig. 4

	Fwd	Rev
Dlk1	TGTGCCAGAGCAGTGCTACACG	GCCATGGCACAACTACACAGGGT
H19	CGACCACTGAGGCATAGCGGC	TGTCCAAGGGCCAAAGTTCGGG
Nap15L	CATGGCCGACCCCGAGAAGC	CGGCATCTTCGCCACCCTGG
Peg3	CACCGCCACTGCGGCAAAAC	GGCTGGCAGGGTCTTCGCAA
Plagl1	CCAGCGAGGGCTGAGTTGCC	GGCCTGGAAGGAAGCGTGCA
IL3ra	TCCCACCCTCGCGATGTGACT	GCACAAACCCTCATGGGGTGGC
Zfp629	TTGGCTCCTGGTGGCGGAGT	GCCCGGAGAGCTCAGGGTGA
Spred2	TGAAGACTACCGGCACGCGC	CCCTTGGCGAAGCGCACGTA
Nefm	TGGGGGCCTCGACTTTGGTCT	CAGGAAGCATCACCGGGCCT
Polrmt	CGGCCGCGGAAGTCCATGTT	CGCAGCGAGGCCCTGTATCG
β-actin	ACACCCGCCACCAGGTAAGCA	CCTGCAGTGAGGTACTAGCCACGA
GAPDH	TTGCTGTTGAAGTCGCAGGAGAC	GGCATCTTGGGCTACACTGAGGAC

	Fwd	Rev
Igf2r_Me	CCAAAGGTTCGGAGGGTTTT	ACTTGGCATAACCAGAATCACAG
Nap1L5_Me	GCAGGGGCTTGTAGATATCGTT	AGAGCTTGCCCAATTCCGTG
Peg13_Me	ATCGTCTACATAGCACCAGCG	TCTGTGACCACGAACCGAAG
Peg3_Me	TAGTGCACCCACACTGAACC	GACGAGCATCGGAGGAGAAG
β-actin	ACACCCGCCACCAGGTAAGCA	CCTGCAGTGAGGTACTAGCCACGA
Nap1L5 NoHpaII	CATGGCCGACCCCGAGAAGC	CGGCATCTTCGCCACCCTGG

Table S4

Related to Fig. 5

A

			Mapped Reads	Total mapped reads
PolyA +	AdGFP	Fwd reads	15042599	31835329
		Rev reads	16792730	
	AdCre	Fwd reads	12668012	25864638
		Rev reads	13196626	
PolyA -	AdGFP	Fwd reads	9242629	19867417
		Rev reads	10624788	
	AdCre	Fwd reads	9299015	19892878
		Rev reads	10593863	

B

1500 random genes		No. of Informative Genes	Total Informative Reads	Reads Mapped to SV129 Maternal Genome	Reads Mapped to Castaneus Paternal Genome
PolyA+	WT (AdGFP)	452	111323	56855	54468
	ATRX KO (AdCre)	438	109409	56352	53057
PolyA-	WT (AdGFP)	261	40432	22223	18209
	ATRX KO (AdCre)	253	38138	20695	17443

C

	Genes with >50 informative SNP reads	Genes with >66% expected parental skewing	Genes with moderate skewing towards expected allele (>60% and <66%)	Genes skewed against expected parental allele	Biallelic Expression
PolyA +	33	12	4	3	14
PolyA -	26	11	1	4	10

Table S5
Related to Fig. 2-4

	**Series**	**Sample ID**	**Contact name**	**Cell type**	**Antibody**
**ATRX**	GSE22162	GSM551138	Richard J Gibbons	Mouse ES	ATRX
**H3K9me3**	GSE57092	GSM1375155	Sarah Diehl	Mouse ES	H3K9me3
**H4K20me3**	GSE26680	GSM656527	Richard A Young	Mouse ES	H4K20me3
**DNA methylation**	GSE28254	GSM821397, GSM821398	Arjen Brinkman, Stunnenberg HG	Mouse ES	Medium and high MethylCap
**H3K4me3**	GSE12241	GSM307618	Tarjei S Mikkelsen	Mouse ES	H3K4me3
**ESHyb H3K4me3**	GSE12241	GSM307605	Tarjei S Mikkelsen	Mouse ESHyb	H3K4me3
**ESHyb H3.3**	GSE16893	GSM487542	Aaron D Goldberg	Mouse ESHyb	HA (H3.3)

Table S1, Related to Figure 2. Features associated with all ATRX bound CGIs in mouse ES cells. ATRX CGIs located within 1 kb of the TSS of a known gene were annotated as promoters. ATRX CGIs which overlapped a known gene but located greater than 1 kb away from a TSS were annotated as intragenic. ATRX CGIs associated with imprinted genes or known monoallelic genes are also shown.

Table S2, Related to Figure 3. List of allelic ChIP primers used in this study.

Table S3, Related to Figure 4. List of qRT-PCR ChIP and DNA methylation primers used in this study.

Table S4, Related to Figure 5. Data related to allelic RNA-seq. A) Total mapped reads in PolyA+ and PolyA- RNA-seq. **B)** Informative SNP reads mapped to SV129 and Castaneus genomes across 1500 random genes. **C)** Allelic expression skewing in imprinted genes with greater than 50 informative SNP reads.

Table S5. List of GEO datasets used throughout this study.

Supplemental Experimental Procedures

ChIP-seq Analysis

Random genomic fragments were generated using Random Genome Fragments on RSA-tools (Thomas-Chollier et al., 2011) using ATRX peaks as a template. Histone modifications were quantitated under random genomic fragments in Seqmonk after normalising for read count and fragment length. Regions were considered enriched for a particular modification if ChIP reads exceeded read counts of the 75th percentile of random fragments. Genome annotations (CGI, TR, ERV) were downloaded from the UCSC Table Browser (mm9 build) (http://genome.ucsc.edu/) (Karolchik et al., 2004) and overlaps were performed in Seqmonk. Where peaks overlapped with multiple features, regions were preferentially annotated in descending order as CGIs, TRs and ERVKs.

DNA Methylation Assay

Genomic DNA was extracted from WT and ATRX KO mouse ES cells and digested with either Msp I or Hpa II, a methylation sensitive isoschizomer. DNA digested with Hpa II were subjected to qRT-PCR with primers which flank an Msp I/Hpa II recognition site at imprinted DMRs. Undigested genomic DNA was combined with Msp I digested DNA in known ratios to obtain a standard curve. Hpa II digested products were plotted against respective curves and normalised to an amplicon which lacked an Msp I/Hpa II recognition site to obtain a methylation ratio. A list of primers used for this study can be found in Table S3.
